# Advancing *Trypanosoma cruzi* N-myristoyltransferase as a drug target for Chagas disease through *in silico* discovery and biochemical evaluation

**DOI:** 10.3389/fmolb.2025.1666768

**Published:** 2026-01-06

**Authors:** Diana González García, Angel Torres, Alan Talevi, Lucas N. Alberca, Miguel A. Beltran, Frida Lara, Marina Da Silva Ferreira, Priscila S. G. Farani, Igor C. Almeida, Rosa A. Maldonado

**Affiliations:** 1 Department of Biological Sciences, The University of Texas at El Paso, El Paso, TX, United States; 2 Laboratory of Bioactive Compounds Research and Development (LIDeB), Faculty of Exact Sciences, National University of La Plata (UNLP), La Plata, Buenos Aires, Argentina; 3 Department of Pharmaceutical Sciences, School of Pharmacy, The University of Texas at El Paso, El Paso, TX, United States

**Keywords:** Chagas disease, *Trypanosoma cruzi*, inhibition, N-myristoyl-transferase, drug targets

## Abstract

**Introduction:**

*N-myristoylation* is a crucial lipid modification that governs protein localization, intracellular trafficking, and function in eukaryotic cells. The enzyme *N-myristoyltransferase* (NMT), which catalyzes this modification, has emerged as an attractive drug target for parasitic diseases. In this study, we performed a comprehensive biochemical and antiparasitic evaluation of *Trypanosoma cruzi* NMT (TcNMT), utilizing novel “*in silico*–identified inhibitors” to assess its potential as a therapeutic agent for Chagas disease.

**Methods:**

Recombinant TcNMT was cloned, expressed, and purified for enzymatic characterization. Catalytic activity and substrate affinity were evaluated using a fluorescence-based assay. Four in-silico-selected NMT inhibitors were screened for (i) enzyme inhibition, (ii) cytotoxicity in human cardiomyocytes, and (iii) antiparasitic activity in *T. cruzi*–infected cardiomyocytes. QUINE and the reference inhibitor DDD85646 were further characterized by calculating selectivity indices. Proteomic profiling of myristoylated proteins was conducted in amastigotes and trypomastigotes following treatment with DDD85646 to identify pathway-level effects.

**Results:**

All recombinant TcNMT preparations were catalytically active and displayed high affinity for peptide substrates. Among the screened compounds, QUINE showed moderate antiparasitic efficacy but very low cytotoxicity, yielding a high selectivity index (SI = 28.11). In contrast, DDD85646 exhibited greater antiparasitic potency but substantially higher host-cell toxicity (SI = 4.67). Proteomic analysis of DDD85646-treated parasites revealed downregulation of myristoylated proteins in both life stages, including ARF GTPases and enzymes associated with vesicular trafficking and lipid metabolism. Host cell proteomes remained largely unchanged.

**Discussion:**

Biochemical characterization and phenotypic testing support TcNMT as a viable therapeutic target for Chagas disease. QUINE demonstrates the most favorable pharmacological profile, combining antiparasitic activity with excellent selectivity and low host toxicity, making it a strong lead candidate for future drug optimization. Proteomics data indicate that NMT inhibition disrupts critical pathways required for parasite viability yet spares host cellular machinery, reinforcing the mechanistic selectivity of TcNMT targeting. Further studies are warranted to improve potency and evaluate in vivo efficacy.

## Graphical Abstract

**Figure:**
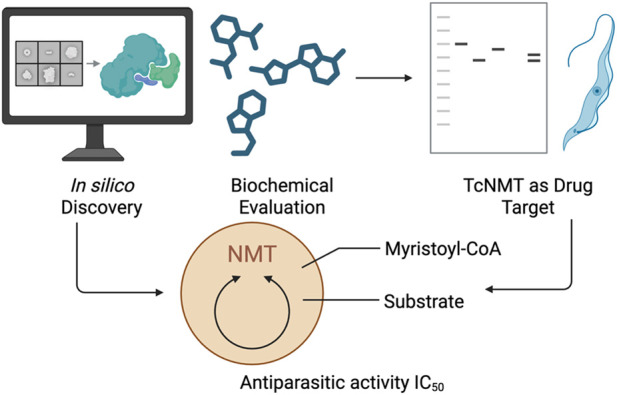
Summary of the discovery and validation of *Trypanosoma cruzi* N-myristoyltransferase (TcNMT) inhibitors, highlighting *in silico* screening, biochemical and cellular evaluation, and proteomic disruption of parasite myristoylated proteins.

## 1 Introduction

Chagas disease, caused by the protozoan parasite *T. cruzi (Trypanosoma cruzi)*, affects an estimated six million people worldwide and represents a major neglected tropical disease burden, particularly in Latin America (Rassi et al., 2010; Rassi et al., 2012). The disease progresses from an acute phase (often asymptomatic or presenting with nonspecific symptoms) to a chronic phase, which may remain indeterminate for decades or evolve into severe cardiac or digestive complications in up to 30%–40% of infected individuals (Rassi et al., 2017). Current treatments rely on benznidazole and nifurtimox, which have established effectiveness during the acute stage but exhibit limited efficacy in chronic infections, commonly associated with high toxicity and poor patient adherence (Bern, 2011; Farani et al., 2024). These limitations underscore the need for novel therapeutic strategies targeting parasite-specific molecules essential to survival and pathogenicity.

One promising molecular target is *T. cruzi* N-myristoyltransferase (TcNMT), an enzyme responsible for the covalent attachment of myristic acid to the N-terminal glycine of specific proteins (Price et al., 2003; Alonso et al., 2019). This lipid modification, known as N-myristoylation, is critical for protein-membrane interactions, intracellular trafficking, signaling, and protein stability. TcNMT follows an ordered Bi-Bi kinetic mechanism, where binding of myristoyl-CoA induces conformational changes in the enzyme that enable subsequent peptide substrate binding and catalysis (Roberts et al., 2014). Importantly, TcNMT shares only moderate sequence identity with its human homologs, supporting the feasibility of selective inhibition. Inhibitors originally developed for *Trypanosoma brucei* NMT, such as DDD85646, have shown promising activity against *T.* cruzi (Herrera et al., 2016), suggesting the conserved enzymatic mechanism can be exploited for chemotherapeutic development (Frearson et al., 2010).

Despite prior validation of TcNMT as a drug target by our laboratory (Herrera et al., 2016), critical knowledge gaps remain regarding its biochemical properties, substrate specificity, inhibitor efficacy, and the downstream effects of NMT inhibition on *T. cruzi* viability and infectivity (Price et al., 2003). In particular, the impact of NMT inhibition on intracellular amastigotes has not been comprehensively investigated. This study addresses these gaps by expressing and purifying recombinant TcNMT, characterizing its enzymatic activity, and evaluating the potency and selectivity of *in silico*–identified inhibitors. We further examined the cytotoxicity of lead compounds in human cardiomyocytes and their antiproliferative activity against intracellular amastigotes and trypomastigotes. Collectively, these analyses clarify the functional role of TcNMT in parasite survival and host infection, reinforcing its potential as a therapeutic target for Chagas disease.

## 2 Materials and methods

### 2.1 Parasite strain and cell culture

*Trypanosoma cruzi* parasites (CL Brener, TcVI) were maintained under standard culture conditions. Epimastigotes were cultured in liver infusion tryptose (LIT) medium at 28 °C, while trypomastigotes were harvested from infected LLC-MK2 cells maintained in DMEM, depending on experimental requirements (Camargo, 1964). Human cardiomyocyte AC16 cells (Thermo Fisher Scientific, Cat# CRL-2920) were cultured in DMEM/F12 supplemented with 5% fetal bovine serum (FBS) and maintained at 37 °C in a humidified incubator with 5% CO_2_ (Davidson et al., 2005).

### 2.2 Recombinant TcNMT cloning, expression and purification

**FIGURE 1:**
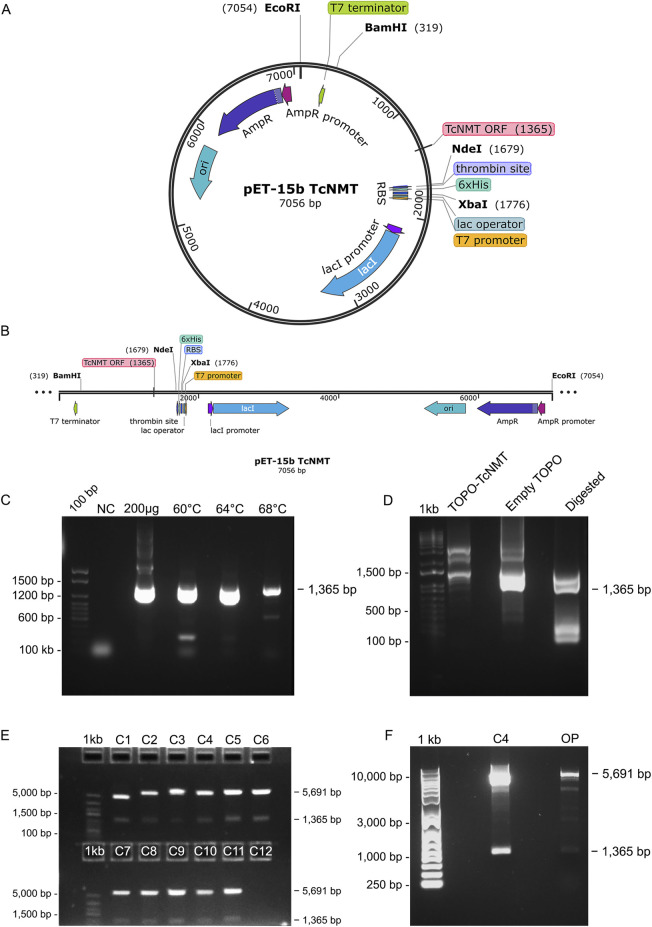
Cloning strategy and validation of the TcNMT expression construct in the pET-15b vector. **(A)** Map of the recombinant plasmid pET-15b/TcNMT (7056 bp), showing the insertion of the 1365 bp TcNMT open reading frame (ORF) between NdeI and BamHI restriction sites. Key annotated features include the T7 promoter, lac operator, ribosome binding site (RBS), thrombin cleavage site, 6xHis tag, and the ampicillin resistance gene (AmpR). **(B)** A linear representation of the construct is shown below the map. **(C)** PCR amplification of TcNMT from *Trypanosoma cruzi* CL Brener genomic DNA using an annealing temperature gradient (60 °C, 64 °C, and 68 °C). A clear 1365 bp band corresponding to the expected ORF was observed at all temperatures, with optimal specificity at 64 °C. NC = no-template control. DNA ladder: 100 bp DNA Ladder (Invitrogen, Cat. No. 15628019). **(D)** Restriction digestion analysis of TOPO: TcNMT and empty TOPO vectors using EcoRI and XbaI. The undigested TOPO: TcNMT plasmid is shown alongside the digested TOPO: TcNMT sample, which released the expected 1,365 bp insert. DNA ladder: E-Gel 1 Kb Plus DNA Ladder (Invitrogen, Cat. No. 10488090). **(E)** Screening of plasmid DNA extracted from colonies C1–C12 by NdeI and BamHI digestion. Positive colonies displayed two bands corresponding to the TcNMT insert (1,365 bp) and vector backbone. Colony 4 (C4) showed a clear, specific insert band and was selected for further propagation. DNA ladder: E-Gel 1 Kb Plus Express DNA Ladder (Invitrogen, Cat. No. 10488091). **(F)** Final validation of colony 4 (C4) by BamHI/XbaI digestion prior to sequencing. The digested C4 plasmid shows the expected two-band pattern, including the released 1,365 bp insert and vector backbone. OP = original, undigested pET-15b plasmid used for transformation. The release of the expected insert confirmed proper construction of the expression plasmid. DNA ladder: GeneRuler 1 Kb DNA Ladder (Thermo Fisher Scientific, Cat. No. SM0312).

The *T. cruzi* N-myristoyltransferase (TcNMT) open reading frame (TcNMT; TriTrypDB accession TcCLB.511283.90) was retrieved directly from TriTrypDB. Gene-specific primers incorporating NdeI and XhoI restriction sites, TcNMT-pET15b-Thrombin_F and TcNMT-pET15b-Thrombin_R were designed from this sequence. Genomic DNA was isolated from CL Brener epimastigotes, and the 1.3 kb TcNMT ORF was amplified using Pfu DNA polymerase (Promega Corporation, Madison, WI, United States) under the following conditions: initial denaturation at 95 °C for 2 min; followed by 30 cycles of denaturation at 95 °C for 30 s, annealing at 58 °C for 30 s, and extension at 72 °C for 1.5 min; with a final extension at 72 °C for 10 min. PCR and digestion products were analyzed by agarose gel electrophoresis alongside the appropriate DNA size markers, as follows: 100 bp DNA Ladder (Invitrogen, Cat. No. 15628019) for Figure 1C, E-Gel 1 Kb Plus DNA Ladder (Invitrogen, Cat. No. 10488090) for Figure 1D, E-Gel 1 Kb Plus Express DNA Ladder (Invitrogen, Cat. No. 10488091) for Figure 1E, and GeneRuler 1 Kb DNA Ladder (Thermo Fisher Scientific, Cat. No. SM0312) for Figure 1F. The PCR product was purified, ligated into the Zero Blunt TOPO vector (Invitrogen (Thermo Fisher Scientific), Carlsbad, CA, United States), and the insert was verified by Sanger sequencing. The confirmed TcNMT fragment was excised with NdeI and XhoI and sub-cloned into the identically digested pET-15b (Novagen (Merck KGaA), Darmstadt, Germany) expression vector, yielding pET15b-Thrombin-TcNMT.

This construct features an N-terminal His_6_ tag, followed by a thrombin cleavage site, upstream of TcNMT for subsequent recombinant expression and affinity purification. Plasmids were transformed into *Escherichia coli* Rosetta-Gami B (DE3) (Novagen (Merck KGaA), Darmstadt, Germany) cells, and protein expression was induced with 1 mM isopropyl β-D-1-thiogalactopyranoside (IPTG) at 18 °C for 16 h. Cells were harvested by centrifugation and lysed via sonication in lysis buffer (25 mM Tris, 500 mM NaCl, 25 mM imidazole, 1 mM TCEP [tris-(2-carboxyethyl)phosphine]/HCl, pH 8.5, DNAse I (Sigma-Aldrich (Merck KGaA), St. Louis, MO, United States) and complete EDTA-free protease inhibitors (Roche Diagnostics GmbH, Mannheim, Germany). The clarified lysate was purified by affinity chromatography using nickel-nitrilotriacetic acid (Ni-NTA) affinity column, and the His-tagged TcNMT protein was eluted with an imidazole gradient. Eluted fractions were pooled and further purified by anion exchange chromatography (HiTrap Q HP, Cytiva (GE Healthcare Life Sciences), Uppsala, Sweden) and size exclusion chromatography (HiLoad 16/600 Superdex 200, Cytiva) (Roberts et al., 2014). Protein concentration was determined using a BCA assay, and purity was confirmed by SDS-PAGE and Western blot with anti-His antibody. The identity of the purified protein was validated by MALDI-TOF (MALDI 8020; Shimadzu Corporation, Kyoto, Japan) mass spectrometry (Frey et al., 2018).

### 2.3 Enzymatic activity assay

TcNMT activity was quantified using a 7-diethylamino-3-(4-maleimido-phenyl)-4-methylcoumarin (CPM) CPM-based fluorescent assay to detect free coenzyme A (CoA) released during the myristoylation reaction (Goncalves et al., 2012). Briefly, 2 µM of recombinant TcNMT was incubated with varying concentrations of Myristoyl-CoA or Hspp60^src^_(2–9)_ substrate in reaction buffer (50 mM Tris-HCl, pH 7.5, 0.5 mM EDTA, 0.1% Triton X-100) containing 100 µM CPM dye. Fluorescence was monitored over time at excitation/emission wavelengths of 380/470 nm using a Cytation 7 multimode plate reader (Agilent). Reaction velocities were determined from the linear portion of the fluorescence curve. Michaelis-Menten parameters (Km and Vmax) were calculated using nonlinear regression fitting in GraphPad Prism (v9.5.0).

### 2.4 *In silico* inhibitor selection and molecular docking

Candidate TcNMT inhibitors were selected through ligand-based virtual screening. Briefly, we retrieved a molecularly diverse dataset of 577 compounds previously tested against both trypanosomatid NMT (with available IC_50_) and whole parasites (with available EC_50_). Compounds were labelled “active” if they presented both IC_50_ and EC_50_ below 1 μM, and “inactive” if they presented both IC_50_ and EC_50_ greater than 1 μM. This resulted in a final dataset comprising 223 active compounds and 256 inactive compounds (the dataset is available on request). A representative balanced training set of 224 compounds was sampled using LibraryMCS 17.2.13.0 (ChemAxon). 3,668 conformation-independent molecular descriptors were computed from the dataset using Dragon 6.0 software (Milano Chemometrics). Highly correlated descriptors were disregarded, and feature bagging was used to obtain 50 descriptor subsets with no more than 250 descriptors each. From each of these, a linear classifier was obtained using a Forward stepwise variable selection procedure, as implemented in the linear discriminant analysis module of Statistica 8.0 (Statsoft). To validate the model performance, we conducted a retrospective virtual screening campaign on a relatively small number of active compounds (112) that were not included in the training set were dispersed across a large number of putative inactive compounds (synthetic decoys, 8548 compounds) obtained from the Enhanced Directory of Useful Decoys resource (Mysinger et al., 2012). Finally, the score of the five models with the best performance in the retrospective screening experiment were combined into a meta-classifier using five combination schemes: MIN operator, which take as MIN score the minimum score across the five combined individual models; MAX operator, which take as MAX score the maximum score across the five combined individual models; AVERAGE operator; which returns as average score from the five models; average RANKING, which first ranks the compounds according to the score obtained from each combined individual model and then uses the average rankings as final score; and VOTING as defined by Zhang and Muegge (2006). The MIN meta-classifier obtained the best average and early enrichment metrics in the retrospective screening (Truchon and Bayly, 2007): Area under the ROC curve of 0.977 and BEROC (alpha = 20) of 0.7481. This model ensemble was used in the prospective virtual screening of DrugBank 4.0 database, an online repository focused on drug repurposing candidates (experimental, approved, shelved and withdrawn drugs) (Law et al., 2014).

Ligand-based models were complemented with molecular docking studies, as this approach can provide further reliability in the predictions of the ligand-based approximations and also hypotheses on the molecular basis of ligand-target interactions. To choose an optimal docking protocol, the orthologous protein of *Leishmania major,* whose experimental structure is available co-crystallized with DDD85646 (PDB ID 2WSA), was initially studied, Both proteins have and acceptable sequence similarity (global identity percentage of 61.39% and identity percentage in the DDD85646 binding pocket of 77.08%) (Supplementary Figure S6).

Scripts from the *molscrub* and *Meeko* repositories of the ForliLab group (https://github.com/forlilab) were employed for the preparation of the ligands and receptors used. *scrub.py* and *mk_prepare_ligand.py*, were used to add spatial coordinates, rotatable bonds, protonation state at pH = 7.4, Gasteiger partial charges and to perform conformational optimization of the ligands (MMFF94 force field, 2,500 steps). The UCSF Chimera 1.16 software was used to remove water molecules and associated ligands, and to add polar hydrogens. Gasteiger partial charges were added and the *.pdbt* file was generated with *mk_prepare_receptor.py*. Finally, energy maps were generated using *autogrid4* from the AutoGrid repository of the Center for Computational Structural Biology (https://github.com/ccsb-scripps/AutoGrid), Docking was performed with AutoDock Vina (Eberhardt et al., 2021), using an exhaustiveness of 128 and generating 10 poses. The grid box was centered at X = 26, Y = 4, Z = 14, with dimensions of 30 × 30 × 30 Å, encompassing the entire ligand binding site. The sequence A0A2V2XL69_TRYCR, corresponding to the NMT of *T*. *cruzi* was obtained from Uniprot https://www.uniprot.org/uniprotkb/A0A2V2XL69/entry) and uploaded to the AlphaFold server (Jumper et al., 2021), without ligands. AlphaFold produced four models; the best of them showed overall good quality, with an ipTM of 0.88 and only a few regions with low confidence (see Supplementary Figures S7, S8). The site where the NMT inhibitor binds was modeled with very high confidence. Three *in silico* hits, Dicyclomine (DICY), Danazol (DANA), and Quinestrol (QUINE) at >98% purity were acquired from Sigma Aldrich. The reference trypanosome inhibitor DDD85646 was purchased from Cayman Chemical Co. (Cat No. 13839). All the compounds were dissolved in DMSO to prepare 1 mM working stocks.

### 2.5 Cytotoxicity assays in human cardiomyocytes

Human cardiomyocyte AC16 cells were seeded at 1 × 10^4^ cells per well in 96-well plates and incubated for 24 h at 37 °C in a humidified incubator with 5% CO_2_. Cells were treated with 2-fold dilutions ranging from 100 to 0.195 μM of each compound and incubated for 72 h. Following treatment, cell viability was assessed using Hoechst 33,342 (Thermo Fisher Scientific, Cat# H3570) and propidium iodide (PI; Thermo Fisher Scientific, Cat# P3566) staining. Fluorescence imaging was performed using the ImageXpress Pico system (Molecular Devices), and viable versus dead cell populations were quantified using CellReporterXpress software (Molecular Devices). IC_50_ values were calculated using nonlinear regression analysis in GraphPad Prism (v9.5.0).

### 2.6 Proliferation assays

AC16 cells were infected with *T. cruzi* trypomastigotes (CL Brener strain) at a multiplicity of infection (MOI) of 10:1 and incubated for 24 h at 37 °C in 5% CO_2_. After incubation, non-internalized parasites were removed by gentle washing with phosphate-buffered saline (PBS), and cells were subsequently treated with varying concentrations of inhibitors for 72 h. At the end of the treatment period, cells were fixed with 4% paraformaldehyde and stained with Hoechst 33,342 (Thermo Fisher Scientific, Cat# H3570) and CellMask™ Deep Red Plasma Membrane Stain (Thermo Fisher Scientific, Cat# C10046). Imaging was performed using a Cytation 7 automated imaging system (Agilent), and image analysis was carried out using CellProfiler (v4.2.7) (Stirling et al., 2021) to quantify intracellular amastigotes and host cell nuclei. Parasite load and host viability were normalized to untreated infected controls.

### 2.7 Parasite isolation from infected cultures for proteomic analysis

AC16 human cardiomyocyte cells were maintained under standard culture conditions (37 °C, 5% CO_2_). For infection assays, cells were seeded at 1 × 10^6^ cells in a 150 cm^2^ flask and grown to ∼80% confluence. *Trypanosoma cruzi* (CL Brenner Strain) tissue-culture trypomastigotes were added to the monolayers at a multiplicity of infection (MOI) of 10:1 (parasites:host cell) and were incubated overnight to allow parasite invasion. The following day, cultures were washed with fresh medium to remove any free, non-internalized parasites, and then incubated for an additional 72 h with or without treatment (DDD85646, 0.43 µM), at 37 °C in a humidified 5% CO_2_ atmosphere. This incubation period enabled intracellular amastigote replication and differentiation into trypomastigotes within host cells. After 72 h of infection, the culture supernatants were harvested to collect newly released trypomastigotes. Supernatants were first subjected to low-speed centrifugation (200 × g, 5 min, 4 °C) to remove residual host cells and large debris. The resulting supernatant was then centrifuged at 2000 *g*/4 °C for 10 min to pellet motile trypomastigotes. Pelleted parasites were washed once with phosphate-buffered saline (PBS) and either processed immediately or stored on ice. In parallel, intracellular amastigotes were obtained from the infected cell monolayers. Infected flasks were gently scraped to dislodge cells, which were then mechanically lysed by gentleMACS Dissociators with gentleMACS M Tubes. The lysate was cleared of bulk debris by a brief centrifugation 300–500 × g/4 °C, 5 min, and the supernatant containing amastigotes was carefully collected. To further purify intracellular amastigotes, the suspension was passed through a diethylaminoethyl (DEAE) cellulose anion-exchange column (DE52, Cytiva) equilibrated in PBS. The column was washed with PBS the flow-through containing the amastigotes was collected. Eluted amastigotes were concentrated by centrifugation (2,000–4,000 × g, 10 min, 4 °C) and washed once with PBS. Parasite yields were quantified by hemocytometer count, and purity was verified microscopically.

### 2.8 Metabolic labeling and click-chemistry enrichment of myristoylated proteins

For metabolic labelling of myristoylated proteins, infected AC16 cultures were treated with 50 μM Click-iT myristic acid, azide (12-azidododecanoic acid; Thermo Fisher Scientific) added to the culture medium during the final 16 h of the 72-h infection period. After labelling, cells were washed with PBS and lysed using the Click-iT Lysis Buffer supplemented with protease inhibitors (provided in the Click-iT Protein Enrichment Kit, Thermo Fisher Scientific Cat no. C10416). Lysates were clarified by centrifugation (10,000 × g/4 °C, 5 min) to remove insoluble material. Azide-labeled proteins were enriched by covalent capture on alkyne agarose resin using the Click-iT® Protein Enrichment Kit, according to the manufacturer’s instructions. Briefly, equal amounts of lysate from labeled and control samples were incubated with the alkyne-functionalized resin in the presence of the copper-catalyst solution to facilitate covalent cycloaddition (“click” reaction) between azide-labeled proteins and the resin. After overnight incubation with gentle agitation, the resin was washed extensively with the provided high-stringency buffers to remove non-specifically bound material. Proteins covalently coupled to the resin were reduced (10 mM dithiothreitol, 70 °C, 15 min) and alkylated (40 mM iodoacetamide, RT, 30 min in the dark) on the resin, followed by digestion with sequencing-grade trypsin (Thermo Fisher). Digestion was carried out at 37 °C for 6–18 h, then peptides were collected from the resin supernatant after centrifugation. Peptides from different sample conditions (e.g., enriched myristoylated proteins from infected vs. control cells, or distinct parasite fractions) were chemically labelled with Tandem Mass Tag (TMT) reagents for multiplexed quantitation. We employed the six-plex TMT™ isobaric labelling kit (TMTsixplex™, Thermo Fisher Scientific) following the manufacturer’s protocol. To reduce sample complexity prior to mass spectrometry, the pooled TMT-labelled peptide sample was fractionated by high-pH reversed-phase chromatography using the Pierce™ High-pH Reversed-Phase Peptide Fractionation Kit (Thermo Fisher Scientific). Each fraction was immediately dried in a vacuum concentrator and stored at −20 °C until analysis. Prior to liquid chromatography–tandem mass spectrometry (LC–MS/MS), fractions were reconstituted in water +0.1% formic acid.

### 2.9 Proteomic LC–MS/MS analysis

Tryptic peptides labeled with TMTsixplex reagents (Thermo Fisher Scientific, Waltham, MA, United States) were analyzed using a Vanquish Neo UHPLC coupled to an Orbitrap Exploris 240 mass spectrometer (Thermo Fisher Scientific) operated in data-dependent acquisition mode. Peptides were separated on a 500 mm × 75 µm i.d. C18 column (BioZen Peptide XB-C18, Phenomenex, Torrance, CA, United States) at a flow rate of approximately 300 nL min^-1^ using a 180 min linear gradient from 6% to 35% acetonitrile with 0.1% formic acid. Survey scans were acquired at 120,000 resolution (m/z 350–1200), and the most intense precursors were fragmented by higher-energy collisional dissociation (HCD). Fragment spectra were acquired at 45,000 resolution with dynamic exclusion enabled to minimize repeat sequencing. Instrument performance was verified using standard peptide quality controls, and parameters followed established LC–MS/MS workflows (Bekker-Jensen et al., 2020).

### 2.10 Proteomic data analysis

Raw spectra were processed in Proteome Discoverer v2.5 (Thermo Fisher Scientific) using the Sequest HT algorithm. Searches were conducted against a combined *Homo sapiens* (UniProt UP000005640) and Trypanosoma cruzi (taxonomy ID 5693) protein database supplemented with common contaminants. Carbamidomethylation of cysteine and TMT modifications on peptide N-termini and lysines were set as fixed; oxidation of methionine and N-terminal acetylation variants were variable. Peptide and protein false-discovery rates were controlled at 1% using target–decoy and Percolator filtering. Reporter-ion intensities (m/z 126–131) were extracted, corrected for isotopic impurities, and normalized across samples for relative quantification. Proteins showing significant differential abundance were identified by non-parametric testing with Benjamini–Hochberg correction (q < 0.05). Identifications were further validated in Scaffold DDA v6.5 (Proteome Software Inc., Portland, OR, United States). Candidate N-myristoylated proteins were identified by screening for N-terminal glycine motifs and confirmed by prediction using the ExPASy Myristoylator tool (Swiss Institute of Bioinformatics).

### 2.11 Statistical analysis

All experiments were performed in at least three biological replicates unless otherwise stated. Statistical analyses were conducted using GraphPad Prism (v9.5.0). Dose-response curves and IC_50_ values were determined using nonlinear regression. Comparisons between groups were assessed by one-way ANOVA followed by Tukey’s *post hoc* test. A p-value <0.05 was considered statistically significant.

## 3 Results

### 3.1 Cloning and validation of the pET-15b: TcNMT expression construct

To generate a recombinant expression system for *T. cruzi* N-myristoyltransferase (TcNMT), the full-length open reading frame (1,365 bp) was amplified from CL Brener genomic DNA and subcloned into the pET-15b vector under the control of the T7 promoter, incorporating an N-terminal 6xHis tag for affinity purification. The cloning strategy was adapted from Roberts et al. (2014) with minor modifications to optimize amplification and vector selection (Roberts et al., 2014). The construct design and annotated plasmid map are shown (Figures 1A,B) (Table 1). PCR amplification using a gradient of annealing temperatures (60 °C, 64 °C, and 68 °C) yielded a distinct band of the expected size at all tested conditions, with 64 °C producing the most specific and intense product (Figure 1C). Cloning into the TOPO vector was confirmed by restriction digestion with EcoRI and XbaI, which released the expected 1,365 bp insert fragment (Figure 1D). The insert was cloned in the expression vector pET-15b. Colony screening by NdeI and BamHI digestion of plasmid DNA extracted from transformed *E. coli* revealed that several clones carried the insert, with colony 4 (C4) showing the cleanest digestion pattern (Figure 1E). Final confirmation of insert integrity was performed by digestion with *BamHI* and *XbaI* (Figure 1F). Although the control pET-15b vector (OP) exhibited faint small fragments near 1.3 kb due to internal restriction sites and partial digestion, only the C4 clone produced the expected two-band pattern corresponding to the 1,365 bp insert and ∼5.7 kb vector backbone. Sanger sequencing further confirmed the presence and correct orientation of the TcNMT insert in the pET-15b/TcNMT construct.

**TABLE 1:** Primers used in this study. Sequences complementary to the open reading frame (ORF) are shown in uppercase, while restriction enzyme recognition sites are indicated in lowercase and underlined.

Primer	Sequence
TcNMT-pET15b-Thrombin_F	5′-catATGGCAGAAGAGGGTTCAGGTTTACATCAG-3′
TcNMT-pET15b-Thrombin_R	5′-ggatccCTATAGCATGAACAATCCCACGTCACTTGG-3′

### 3.2 Expression, purification, and mass validation of recombinant TcNMT

**FIGURE 2:**
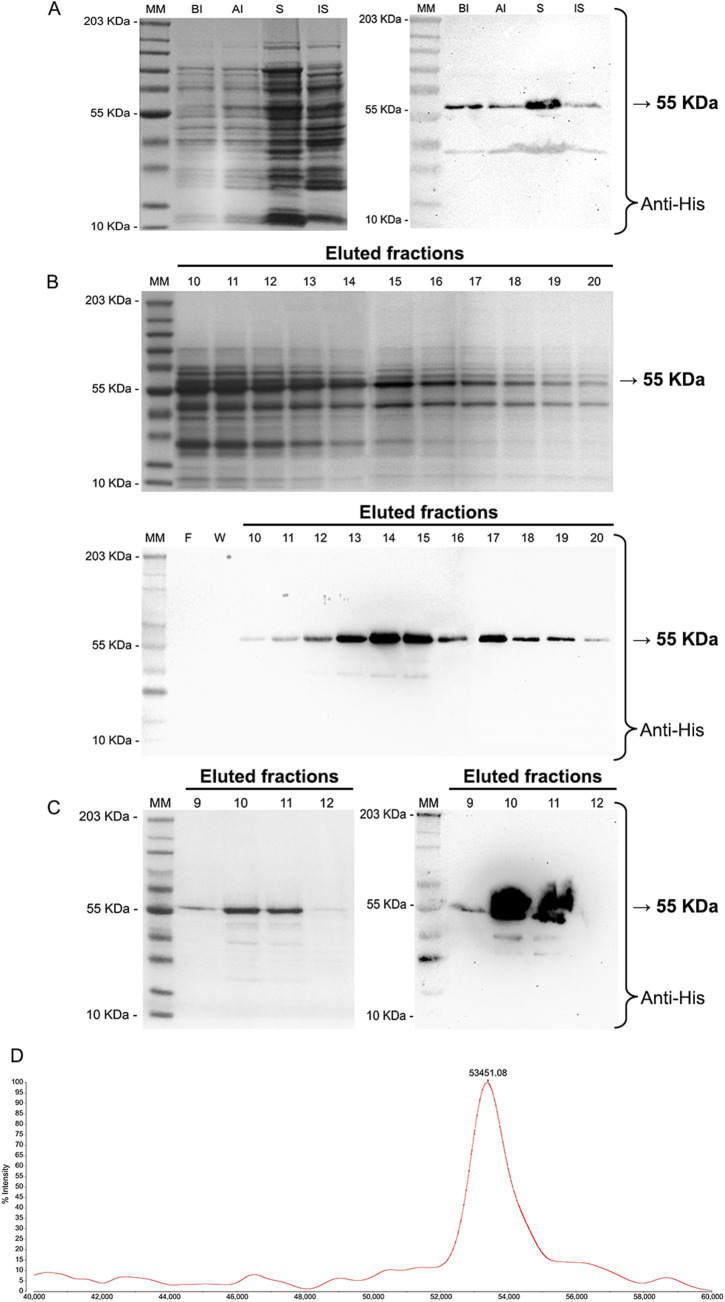
Expression, purification, and validation of recombinant *Trypanosoma cruzi* N-myristoyltransferase (TcNMT). **(A)** SDS-PAGE (left) and Western blot (right) showing total expression and solubility of recombinant TcNMT. BI: bacterial lysate before induction; AI: after IPTG induction; S: soluble fraction. A distinct band at 55 kDa corresponds to the expected molecular weight of TcNMT, confirmed by immunodetection using anti-His antibody. **(B)** Nickel affinity chromatography of the soluble fraction using Ni-NTA resin. Fractions 6–22 were collected and analyzed by SDS-PAGE (top) and Western blot (bottom). The target protein eluted between fractions 11–18 with high purity and strong immunoreactivity at 55 kDa. MM: molecular marker; F: flow-through; W: wash fraction. **(C)** Anion exchange chromatography showing fractions 9–12 on SDS-PAGE (left) and corresponding Western blot (right). The strongest signal for TcNMT was observed in fractions 10–11, consistent with further enrichment. **(D)** MALDI-TOF mass spectrometry analysis of the final purified protein. The main peak at 53,451.08 m/z confirms the expected molecular weight of recombinant TcNMT, validating its identity.

Recombinant TcNMT was expressed in *E. coli* Rossetta-Gami B (DE3) cells and purified using affinity and ion exchange chromatography steps, following the protocol of Roberts et al. (2014) with adaptations to the purification workflow (Roberts et al., 2014). Upon IPTG induction, expression of a 55 kDa band corresponding to TcNMT was detected by SDS-PAGE and confirmed by Western blot analysis using anti-His antibodies (Kamble et al., 2025). The protein was predominantly present in the soluble fraction, indicating efficient folding and solubility under the chosen expression conditions (Figure 2A). Ni-NTA affinity purification yielded protein-rich fractions between lanes 11 and 18, as shown by Coomassie blue staining and immunoblotting (Figure 2B). Further purification by anion exchange chromatography resulted in highly enriched TcNMT in fractions 10 and 11 (Figure 2C), with subsequent completion of sample clean-up by size exclusion chromatography. The identity and integrity of the final purified product were confirmed by MALDI-TOF mass spectrometry, which showed a major peak at 53,451.08 Da, consistent with the expected molecular weight of the His-tagged recombinant TcNMT (Figure 2D).

### 3.3 Biochemical characterization and inhibitor profiling of TcNMT

**FIGURE 3:**
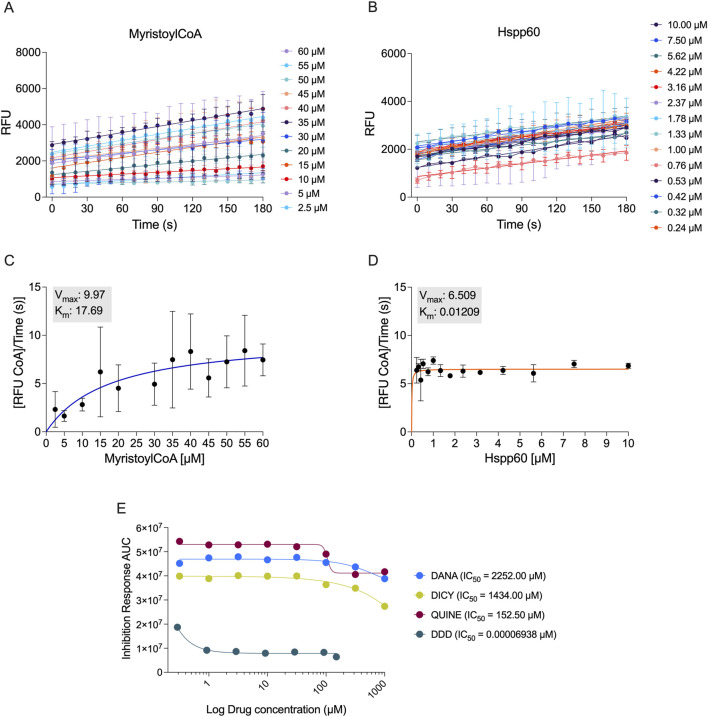
Enzymatic activity characterization of recombinant *Trypanosoma cruzi* N-myristoyltransferase (TcNMT) and inhibitor profiling. **(A)** Fluorescence-based detection of CoA release over time using increasing concentrations of Myristoyl-CoA substrate. **(B)** Fluorescence-based detection of CoA release with increasing concentrations of Hspp60 protein substrate. **(C)** Michaelis-Menten plot derived from Myristoyl-CoA titration, with calculated kinetic parameters: Km = 17.69 µM and Vmax = 9.97 RFU CoA/time (s). **(D)** Michaelis-Menten plot for Hspp60 substrate with Km = 0.01209 µM and Vmax = 6.509 RFU CoA/time (s). **(E)** Inhibitory activity of four in silico-designed compounds against TcNMT, assessed using a CPM-CoA fluorescence assay. Dose-response inhibition curves were used to calculate IC_50_ values: DANA (IC_50_ = 2252.00 µM), DICY (IC_50_ = 1434.00 µM), QUINE (IC_50_ = 152.50 µM), and DDD85646 (IC_50_ = 0.00006938 µM), a total of *n = 16 data points* were included in each analysis, derived from multiple replicates across independent experiments. All assays were conducted in optimized reaction conditions using purified recombinant TcNMT. Data represents mean ± standard deviation (SD) of three technical replicates from three independent biological experiments. Statistical analysis was performed using One-way ANOVA.

To evaluate the enzymatic properties of *T. cruzi* N-myristoyltransferase (TcNMT), we first optimized an *in vitro* fluorescence-based assay using the CPM reagent to detect CoA release during the myristoylation reaction. TcNMT activity was assessed using titrations of Myristoyl-CoA and Hspp60 as donor and acceptor substrates, respectively. Increasing concentrations of Myristoyl-CoA produced a time-dependent increase in fluorescence (Figure 3A), and kinetic analysis yielded a Michaelis-Menten constant (Km) of 17.69 µM and a maximum velocity (Vmax) of 9.97 RFU CoA/time (s) (Figure 3C). Similarly, Hspp60 substrate titration resulted in a concentration-dependent increase in fluorescence (Figure 3B), with the corresponding Michaelis-Menten plot revealing a Km of 0.01209 µM and a Vmax of 6.509 RFU CoA/time (s) (Figure 3D). These results confirm that recombinant TcNMT is catalytically active and capable of transferring myristoyl groups to protein substrates.

Next, we assessed the ability of four compounds to inhibit TcNMT activity using the CoA-release assay. Dose–response inhibition curves were generated for each compound, revealing that DDD85646 exhibited the highest potency, with an apparent IC_50_ of 0.00006938 µM (Figure 3E). We note, however, that the DDD85646 curve did not display a classical sigmoidal profile with a clear upper plateau, reflecting the very high potency of this compound at sub-nanomolar concentrations. Consequently, the reported IC_50_ should be considered an approximation within the concentration range tested, and future studies including lower concentrations will be required for a more precise determination. In contrast, DICY, DANA, and QUINE displayed higher IC_50_ values of 1434.00 µM, 2252.00 µM, and 152.50 µM, respectively. These data confirm that DDD85646 is a highly potent inhibitor of TcNMT, supporting its further evaluation in cellular infection models.

### 3.4 Cytotoxicity of TcNMT inhibitors in human cardiomyocytes

To assess the cytotoxicity of TcNMT inhibitors in host cells, AC16 human cardiomyocytes were treated with increasing concentrations of DANA, DICY, QUINE, and DDD85646 (DDD) for 72 h. Cell viability was determined using a standardized dual-staining assay with Hoechst 33,342 and propidium iodide, which directly distinguishes live (blue) from dead (red) cells based on nuclear and membrane integrity. High-content fluorescence microscopy was coupled with automated image analysis to ensure objective and quantitative assessment of cell viability. Viability data were expressed relative to untreated controls, and dose–response curves were generated to calculate IC_50_ values.

**FIGURE 4:**
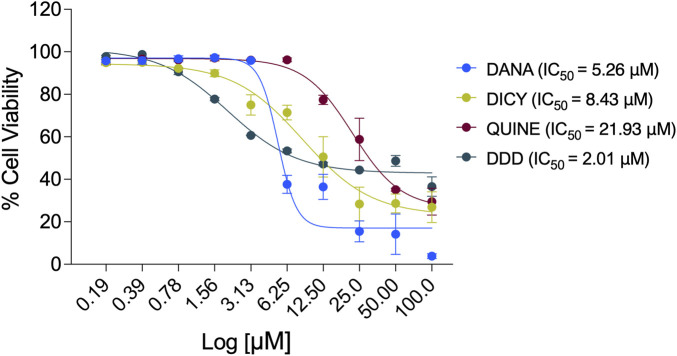
Cytotoxicity assessment of “*in silico*-identified TcNMT inhibitors” in human cardiomyocytes. Dose-response curves show the effect of increasing concentrations of four TcNMT inhibitors DANA, DICY, QUINE, and DDD85646 (DDD), on AC16 human cardiomyocyte viability after 72 h of treatment. Cell viability was determined using a fluorescence-based assay and is expressed as a percentage relative to untreated controls. DDD exhibited the highest cytotoxicity with an IC_50_ of 2.01 µM, followed by DANA (IC_50_ = 5.26 µM), DICY (IC_50_ = 8.43 µM), and QUINE (IC_50_ = 21.93 µM). Data points represent the mean ± standard deviation (SD) of three technical replicates. Nonlinear regression analysis was performed to calculate IC_50_ values.

Dose-response analysis (Figure 4) indicated that DDD exhibited the greatest cytotoxicity (IC_50_ = 2.01 µM), while DICY (IC_50_ = 8.43 µM), DANA (IC_50_ = 5.26 µM), and QUINE (IC_50_ = 21.93 µM) were markedly less toxic. Quantitative imaging (Supplementary Figure S1) revealed a concentration-dependent increase in cell death, particularly notable for DDD at concentrations ≥1.56 µM. Benznidazole (Bz), included as a control at its standard treatment concentration (100 µM), demonstrated minimal cytotoxic effects.

These results confirm DDD as the most cytotoxic compound among the TcNMT inhibitors tested, while DANA, DICY, and QUINE, although they did not confirm significant inhibition of the recombinant TcNMT, exhibited more favorable host cell viability profiles across a broader concentration range, in line with would be expected from repurposing drugs.

### 3.5 Inhibition of *Trypanosoma cruzi* infection and intracellular replication by TcNMT inhibitors

**FIGURE 5:**
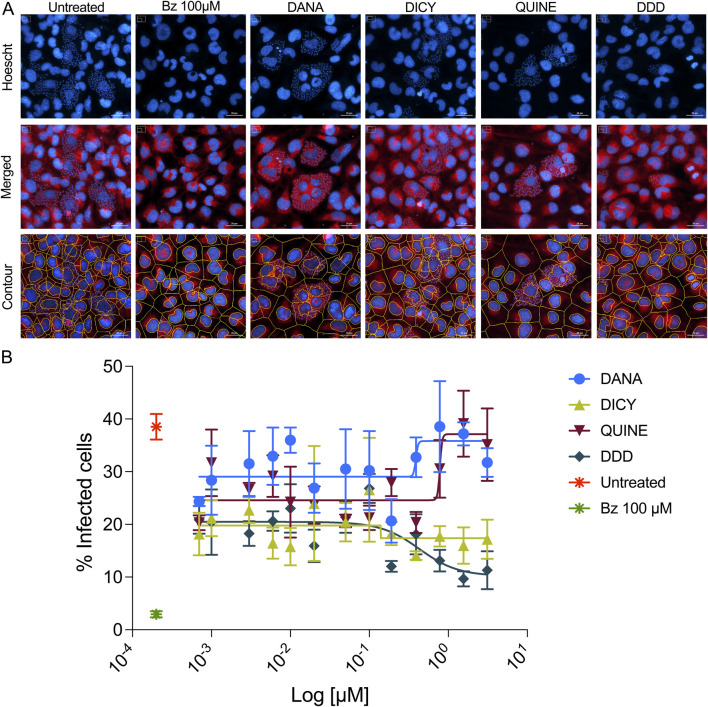
Inhibition of *Trypanosoma cruzi* infection in AC16 human cardiomyocytes by TcNMT inhibitors. **(A)** Representative high-content fluorescence microscopy images of AC16 cells infected with *Trypanosoma cruzi* (CL Brener strain) and treated for 72 h with either vehicle (untreated), Bz (100 μM), or one of four TcNMT inhibitors (DANA, DICY, QUINE, or DDD85646). Nuclei were stained with Hoechst 33,342 (blue), and host cell membranes with CellMask Deep Red (red). Intracellular parasites appear as smaller Hoechst-positive puncta within the host cytoplasm. The bottom row shows CellProfiler-generated segmentation masks overlaid on merged images. Images were acquired using a Cytation 7 imaging system (BioTek) at ×20 magnification. Scale bar: 30 μm. **(B)** Quantification of infection rates based on high-content image analysis. Data shows the percentage of infected host cells after treatment with serial dilutions of each TcNMT inhibitor or controls. Dose-response curves were generated for each compound and IC_50_ values calculated using nonlinear regression: DANA (0.39 µM), DICY (N/A), QUINE (0.78 µM), and DDD85646 (0.43 µM). Data represents the mean ± standard deviation (SD) of three technical replicates.

To determine the antiparasitic efficacy of TcNMT inhibitors, AC16 human cardiomyocytes were infected with *T*. *cruzi* (CL Brener strain) and treated for 72 h with vehicle control, Bz (100 μM), or increasing concentrations of the *in silico*-identified TcNMT inhibitors DANA, DICY, QUINE, and DDD85646 (DDD). High-content fluorescence microscopy revealed the intracellular distribution of parasites, visible as small Hoechst-positive puncta within host cell cytoplasm (Figure 5A). Host nuclei and membranes were visualized using Hoechst 33,342 and CellMask Deep Red, respectively, and segmentation masks generated by CellProfiler were used to quantify infection levels. Quantitative image analysis showed a concentration-dependent reduction in the percentage of infected cells for several of the tested compounds (Figure 5B). DDD and DANA displayed the highest potency, with IC_50_ values of 0.43 µM and 0.39 µM, respectively, followed by QUINE with an IC_50_ of 0.78 µM. DICY did not exhibit a consistent dose-dependent effect, and an IC_50_ value could not be determined. These results indicate that DDD, DANA, and QUINE effectively reduce *T. cruzi* infection in host cells, with DDD showing a potent response even at sub-micromolar concentrations.

Further analysis using a direct quantification of infection rate (% infected cells) across a wider concentration range is shown in Supplementary Figure S3. Benznidazole (100 µM) served as a positive control and significantly reduced infection levels compared to untreated cells (Supplementary Figures S2A–D, red vs. purple bars). Among the TcNMT inhibitors, DDD showed the most significant reduction in infection, even at sub-micromolar concentrations (Supplementary Figure S2D), while DICY also showed inhibitory effects at concentrations below 1 µM (Supplementary Figure S2B). QUINE had moderate activity at lower micromolar concentrations (Supplementary Figure S2C), and DANA exhibited minimal reduction in infection at any tested dose (Supplementary Figure S2A).

To assess the impact of the compounds on intracellular parasite replication, the average number of amastigotes per infected cell was quantified (Supplementary Figure S3). Benznidazole and DDD again produced the most consistent reductions across concentrations, with significant effects seen as low as 1.56 µM (Supplementary Figure S3D). DICY (Supplementary Figure S3B) and QUINE (Supplementary Figure S3C) showed moderate reductions at higher concentrations, while DANA had a negligible impact on intracellular replication (Supplementary Figure S3A).

Lastly, we calculated the endocytic index (percentage of infected cells × mean amastigotes per cell) to integrate infection and replication data (Supplementary Figure S4). DDD demonstrated the strongest and most consistent reduction in endocytic index values across its concentration range (Supplementary Figure S4D), similar to benznidazole. DICY (Supplementary Figure S4B) and QUINE (Supplementary Figure S4C) showed partial reductions, particularly at intermediate doses, while DANA (Supplementary Figure S4A) had minimal impact.

To assess compound specificity and therapeutic potential, we calculated the Selectivity Index (S.I.) as the ratio of host cell cytotoxicity (CC_50_) to antiparasitic efficacy (ED_50_) (Table 2). Among the tested compounds, DDD85646 exhibited the strongest *in vitro* enzymatic inhibition of TcNMT, with an IC_50_ of 0.000069 µM. However, its selectivity index was low (S.I. = 4.67), reflecting a narrow therapeutic window due to higher cytotoxicity (CC_50_ = 2.01 µM) in host cells. In contrast, QUINE displayed moderate enzymatic inhibition (IC_50_ = 152.50 µM) but showed the highest S.I. value (28.11) in cell-based assays. This was attributed to its low host cell cytotoxicity (CC_50_ = 21.93 µM) and good antiparasitic efficacy (ED_50_ = 0.78 µM), suggesting a more favorable therapeutic profile. DANA demonstrated moderate antiparasitic activity (ED_50_ = 0.39 µM) and cytotoxicity (CC_50_ = 5.26 µM), resulting in an S.I. of 13.48. DICY, on the other hand, showed poor TcNMT inhibition and no detectable antiparasitic activity. Overall, while DDD85646 remains the most potent TcNMT-targeting compound at the biochemical level, QUINE emerged as the most promising candidate in cellular models, offering the best balance between efficacy and safety.

**TABLE 2:** TcNMT inhibitors activity. CC_50_: concentration that causes 50% cell death or toxicity; IC_50_: half-maximal inhibitory concentration of the enzyme; ED_50_: effective dose that inhibits parasite proliferation by 50% relative to an untreated control; SI: Selectivity index, the ratio of a drug’s toxicity to its efficacy.

Compound	AC16 cells	Recombinant TcNMT	*Trypanosoma cruzi* proliferation assay	
	CC_50_ (μM)	IC_50_ (μM)	ED_50_ (μM)	S.I.
1. DANA 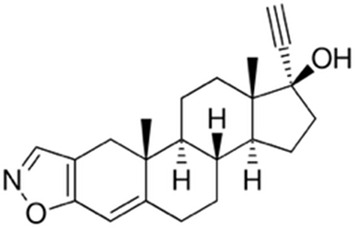	5.26	2252.00	0.39	13.48
2. DICY 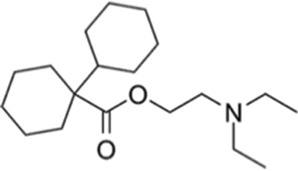	8.43	1434.00	N/A	N/A
3. QUINE 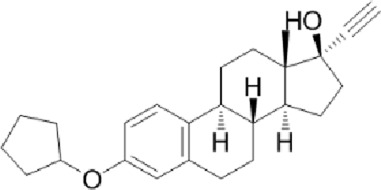	21.93	152.50	0.78	28.11
4. DDD85646 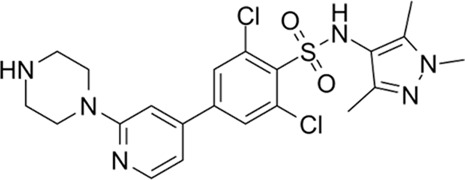	2.01	0.000069	0.43	4.67

### 3.6 Selective disruption of myristoylated proteins by TcNMT inhibition in *Trypanosoma cruzi* and host cells

To determine the global impact of TcNMT inhibition on host and parasite protein expression, we conducted a quantitative proteomic analysis across three biological contexts: uninfected AC16 human cardiomyocytes, intracellular amastigotes, and extracellular trypomastigotes. Proteomic profiles were generated using LC-MS/MS following treatment with the TcNMT inhibitor DDD85646 (control drug), and the resulting data were analyzed for differential expression (Supplementary Table S1).

In uninfected AC16 cells, 90 proteins were significantly upregulated and 132 downregulated (Supplementary Figure S5A; Supplementary Table S2). These changes were modest and primarily involved cytoskeletal and membrane-associated proteins, consistent with a limited off-target effect of the compound on the host proteome. In contrast, intracellular amastigotes showed a marked proteomic response, with 10 proteins upregulated and 76 significantly downregulated upon treatment (Supplementary Figure S5B; Supplementary Table S2). Many of the suppressed proteins in this compartment were involved in fatty acid metabolism, vesicle transport, and signaling pathways, and functional categories dependent on myristoylation. Extracellular trypomastigotes also displayed significant proteomic alterations, with 26 proteins upregulated and 37 downregulated following TcNMT inhibition (Supplementary Figure S5C; Supplementary Table S2). Several of these were conserved hypothetical proteins or enzymes involved in post-translational modifications and membrane dynamics, suggesting a critical role for myristoylation in parasite viability and adaptation.

**FIGURE 6:**
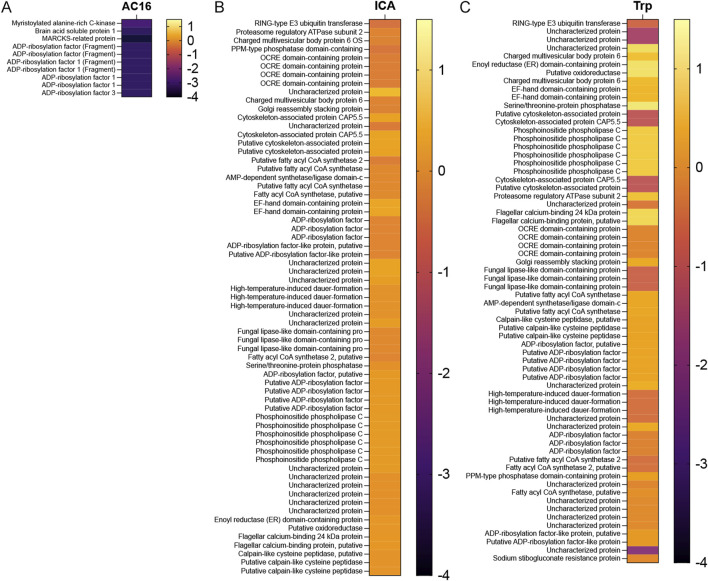
Heatmap analysis of myristoylated proteins following DDD85646 treatment in host cells and different parasite stages. Heatmaps represented in log_2_ fold changes in protein abundance upon treatment with the TcNMT inhibitor DDD85646, as determined by label-free quantitative proteomics. Proteins were selected based on significance thresholds (p < 0.05 and |log_2_ fold change| > 1). **(A)** Myristoylated differential protein expression in uninfected AC16 human cardiomyocytes (AC16). **(B)** Myristoylated differential protein expression in intracellular amastigotes (ICA) isolated from infected AC16 cells. **(C)** Myristoylated differential protein expression in extracellular trypomastigotes (Trp). Each row represents a protein, and color intensity reflects the magnitude of upregulation (yellow) or downregulation (purple) relative to untreated controls. The scale bar ranges from −4 (strong downregulation) to +1 (mild upregulation).

To evaluate the specific impact of TcNMT inhibition on N-myristoylated proteins, we analyzed a curated list of predicted or experimentally validated myristoylated proteins across host and *T. cruzi* compartments (Supplementary Table S3). In uninfected AC16 cardiomyocytes, two host proteins were significantly downregulated: MARCKS (Myristoylated alanine-rich C-kinase substrate; Log_2_FC = −2.97, p = 0.003) and BASP1 (Brain acid soluble protein 1; Log_2_FC = −3.27, p = 0.0068). Both proteins are known to associate with the plasma membrane and regulate cytoskeletal dynamics and intracellular signaling, functions that are critically dependent on N-myristoylation. Their suppression suggests subtle host modulation but confirms a limited off-target effect of TcNMT inhibitor (Figure 6A).

The intracellular amastigotes exhibited selective downregulation of several *T. cruzi* proteins predicted to undergo N-myristoylation, among the most significantly affected were members of the ADP-ribosylation factor (ARF) family, including ARF1 and ARF3 (Log_2_FC = −3.25, p = 0.0418) (Table 2; Supplementary Table S3), which are essential for vesicular trafficking and Golgi structure. These GTPases depend on N-terminal myristoylation for membrane localization and function, suggesting that TcNMT inhibition disrupts vesicle-mediated transport and intracellular organization in the amastigote stage. Additional downregulated myristoylated proteins in ICA included enzymes involved in lipid metabolism and membrane anchoring, such as fatty acyl-CoA synthetases, which are responsible for activating fatty acids for downstream lipid biosynthesis. These proteins are essential for maintaining the lipid-rich intracellular environment that supports parasite replication (Figure 6B).

In trypomastigotes, DDD85646 treatment led to significant downregulation of several parasite myristoylated proteins with roles in post-translational modification, signal transduction, and cytoskeleton-membrane interactions (Supplementary Table S3). Notably, ARF1 and ARF3 isoforms were again found to be significantly suppressed (Log_2_FC = −3.25, p = 0.0418), consistent with their role across parasite stages and confirming the persistent vulnerability of this trafficking machinery to TcNMT inhibition (Table 3). Also downregulated were putative phosphoinositide-processing enzymes and several uncharacterized proteins containing N-terminal glycine residues, hallmarks of canonical myristoylation motifs. While their precise biological roles remain to be defined, their suppression suggests disruption of myristoylation-dependent membrane targeting and protein–protein interactions that facilitate parasite motility and host cell invasion (Table 3). In addition to suppressed targets, we identified one significantly upregulated myristoylated protein calpain-like cysteine peptidase (Log_2_FC = +2.11, p = 0.045) (Table 3). Calpains are calcium-dependent proteases implicated in cytoskeletal remodeling, parasite motility, and host–parasite interactions. The upregulation of this protein may represent a compensatory response to stress or impaired signaling pathways caused by TcNMT inhibition. Alternatively, this increase could reflect a role in parasite differentiation or survival under drug pressure, although its precise function remains to be fully elucidated (Figure 6C).

**TABLE 3:** Differentially expressed predicted myristoylated proteins in DDD85646-treated host (AC16) and *Trypanosoma cruzi* cells identified by proteomic analysis.

Cells	Accession Number	Gene	ID	Organism	P-value
AC16	P29966	MARCKS	Myristoylated alanine-rich C-kinase substrate	*Homo sapiens*	0.0034
	P80723	BASP1	Brain acid soluble protein 1	*Homo sapiens*	0.0068
	A0A8V8TNZ5	ARF1	ADP-ribosylation factor (fragment)	*Homo sapiens*	0.0418
	P61204	ARF3	ADP-ribosylation factor (fragment)	*Homo sapiens*	0.0418
ICA	Tc00.1047053503939.120	Q4DLJ9	RING-type E3 ubiquitin transferase	*T. cruzi*	0.0124
	MOQ_006966	K2N3N6	Proteasome regulatory ATPase subunit 2	*T. cruzi*	0.0343
Tryp	Tc00.1047053503939.120	Q4DLJ9	RING-type E3 ubiquitin transferase	*T. cruzi*	0.0155
	TCDM_02894	V5DLL6	Uncharacterized protein	*T. cruzi*	0.0300
	C3747_33g216	A0A2V2X214	Uncharacterized protein	*T. cruzi*	0.0300
	Tc00.1047053506341.20	Q4CYW5	Uncharacterized protein	*T. cruzi*	0.0321

These findings demonstrate that TcNMT inhibition targets a defined and functionally critical set of parasite myristoylated proteins, resulting in stage-specific proteomic reprogramming that compromises parasite viability and infectivity. Importantly, our methodology also identified the flagellar calcium-binding protein (FCaBP), a protein previously validated as myristoylated in *T. cruzi* (Godsel and Engman, 1999). Although detected here under the accession corresponding to the CL Brener strain, this concordance with prior experimental evidence further validates the specificity and accuracy of our proteomic approach in identifying biologically relevant N-myristoylated targets. Together, these data underscore the stage-specific impact of TcNMT inhibition on the *T. cruzi* myristoylated proteome and further validate this pathway as a selective target for therapeutic intervention.

### 3.7 Structural modeling of tcNMT-Ligand complexes highlights distinct binding modes

**FIGURE 7:**
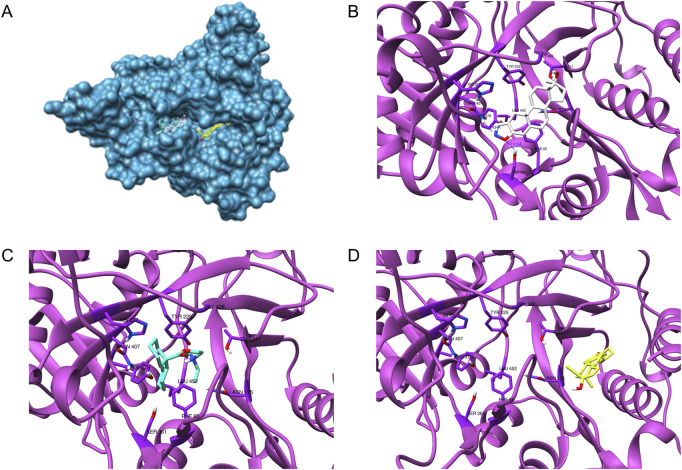
Structural model of *Trypanosoma cruzi* N-myristoyltransferase (TcNMT) in complex with candidate inhibitors. **(A)** Overall cartoon representation of TcNMT (magenta), showing the predicted binding poses of the three lead compounds: DANA (white), DICY (cyan), and QUINE (yellow), docked into the active site using AutoDock Vina. **(B)** Close-up of the DANA binding pose, highlighting hydrogen bond interactions with residues SER361, TYR376, ASN407 (via the nitrogen atom), and ASN429 (via the oxygen atom). DANA also establishes hydrophobic contacts with PHE85, TYR225, PHE240, and LEU430. **(C)** Predicted binding mode of DICY showing hydrophobic interactions with aromatic residues PHE83, PHE85, PHE240, and TYR225 within the TcNMT binding pocket. **(D)** Binding pose of QUINE, illustrating extensive hydrophobic interactions with TRP12, VAL76, ILE174, LEU177, ILE193, TYR210, LEU216, and TYR435.

To evaluate the binding behavior of candidate inhibitors within *T. cruzi* N-myristoyltransferase (TcNMT), we performed a comprehensive structural modeling and docking analysis. We initially validated our docking pipeline using *L. major* NMT (LmNMT) as a reference, which possesses a co-crystallized structure with DDD85646 (PDB ID: 5O2Z). This ortholog presented a reasonable overall sequence identity, and the residues that make up the binding site of that ligand were highly conserved (Supplementary Figure S6), confirming the ability of our docking protocol to predict the ligand pose, with the redocked ligand exhibiting a low RMSD value (<1 Å) compared to the ligand pose in the experimental structure. The best model of TcNMT obtained by AlphaFold showed predominantly very high and high confidence regions, and good overlap with the experimental structure of *Leishmania major* enzyme (Supplementary Figure S7), with strong conservation of the overall fold and the binding pocket architecture (Supplementary Figure S8). Docking of DDD85646 into TcNMT model showed a virtually identical binding pattern compared with the one observed in *L. major* structure (Supplementary Figure S9). Figure 7 depicts TcNMT in complex with our candidate inhibitors (Figure 7A). Interestingly, whereas DANA (Figure 7B) and DICY (Figure 7C) were predicted to bind in the same exact position as DDD85646, QUINE was predicted to bind in an adjacent region (Figure 7D). Taken together, these results underscore both the structural conservation and subtle divergences in binding pocket topology that may affect drug–target interactions across trypanosomatid species. The unique binding pose of QUINE suggests it could serve as a lead for structure-based optimization campaigns targeting novel allosteric or extended substrate sites.

## 4 Discussion

Our findings establish *T*. *cruzi* N-myristoyltransferase (TcNMT) as a critical enzyme for parasite survival and a high-value drug target, in agreement with earlier studies in kinetoplastids (Price et al., 2003; Roberts et al., 2014; Herrera et al., 2016). By cloning and expressing recombinant TcNMT, we confirmed that this enzyme mirrors the essential properties observed in other organisms, as it is expressed as a soluble 55 kDa protein capable of catalyzing the co-translational attachment of myristate to N-terminal glycine residues on substrate proteins (Price et al., 2003). Kinetic analyses demonstrated that TcNMT follows a sequential ordered Bi–Bi mechanism characteristic of NMTs, wherein myristoyl-CoA binding induces an active conformation for subsequent peptide substrate recognition (Rudnick et al., 1991). Consistent with this mechanism, our enzyme assays revealed a moderate affinity for myristoyl-CoA (Km in the low micromolar range) but a strikingly high affinity for the peptide substrate (nanomolar Km), suggesting that once the acyl-donor is bound, TcNMT can efficiently capture and modify its protein target. Such behavior aligns with prior structural and kinetic studies of yeast and human NMTs (Rudnick et al., 1991). Notably, the successful *in vitro* reconstitution of TcNMT activity (validated by CoA release assays and MALDI-TOF mass confirmation of the purified enzyme) provides a solid experimental platform to evaluate inhibitors against this parasite enzyme.

The therapeutic value of these inhibitors was further contextualized by their performance in host cells. DANA, DICY, and QUINE exhibited moderate cytotoxicity in AC16 cardiomyocytes (CC_50_ values between 5 and 22 µM), whereas DDD85646 was more cytotoxic (CC_50_ = 2.01 µM), indicating a narrower therapeutic window. Notably, our proteomic analysis of uninfected AC16 cells treated with DDD85646 revealed reduced abundance of MARCKS and BASP1, canonical host N-myristoylated proteins involved in cytoskeletal dynamics, consistent with partial off-target inhibition of human NMT (Roberts and Fairlamb, 2016). Conversely, the weaker inhibitors showed minimal host proteomic perturbation, consistent with their poor engagement of NMT in both parasite and host. These data underscore the challenge of optimizing NMT inhibitors to maximize parasite selectivity while minimizing host toxicity. Moreover, evaluation of antiparasitic activity in *T. cruzi*-infected cardiomyocytes revealed that DDD85646 significantly reduced parasite burden, with an ED_50_ of 0.43 µM. This efficacy, close to its cytotoxicity threshold, highlights both its potent intracellular action and the need for optimization. QUINE showed moderate activity (ED_50_ = 0.78 µM), while DANA, despite initially promising infection index reductions, did not significantly impair amastigote replication. DICY lacked activity altogether. These differences were reinforced by the proliferation Selectivity Index (S.I.), where QUINE outperformed all compounds (S.I. = 28.11), due to its relatively high host cell tolerance. In contrast, DDD85646, though potent, had a lower S.I. (4.67), indicating its cytotoxic liability (Herrera et al., 2016). Collectively, these results prioritize DDD85646 as the most potent enzyme-targeted molecule, while identifying QUINE as a more balanced lead candidate for therapeutic development. These conclusions align well with previous studies, which found that several DDD85646-like NMT inhibitors could halt intracellular *T. cruzi* proliferation at sub-micromolar doses with minimal host toxicity (Herrera et al., 2016). Notably, DDD85646 was originally developed for *Trypanosoma brucei* infections in which it cured mice at tolerable doses (Frearson et al., 2010; Roberts et al., 2014), so its efficacy in our *T. cruzi* model bolsters the case for repurposing this scaffold for Chagas disease despite the need to fine-tune its selectivity.

To dissect the molecular consequences of TcNMT inhibition, we performed comparative proteomics on host cells and parasites, which yielded insights into why NMT blockade is lethal to *T. cruzi*. We found that DDD85646 treatment selectively downregulated numerous parasite proteins known or predicted to be N-myristoylated, implicating these as critical downstream effectors of the drug. Foremost among these were the ADP-ribosylation factor (ARF) family GTPases ARF1 and ARF3, which showed significant suppression in DDD85646-treated amastigotes and trypomastigotes. ARF proteins are master regulators of vesicular trafficking and Golgi structure; they must be myristoylated at their N-terminus to associate with Golgi membranes and recruit coat proteins for vesicle formation. Thus, inhibiting TcNMT likely mislocalizes ARF1/3 to the cytosol, collapsing the parasite’s secretory pathway and blocking transport of essential cargo. This mechanism is consistent with previous chemoproteomic profiling of *T. cruzi*, which identified ARF1 among the ∼50 proteins comprising the parasite’s N-myristoylome (Roberts and Fairlamb, 2016). Our results directly confirm that interfering with myristoylation destabilizes ARF1/3, providing a tangible link between NMT inhibition and disruption of endomembrane trafficking. In turn, this explains observations from earlier genetic studies where overexpression of NMT in *Leishmania* led to lipid-filled, disordered cells (Price et al., 2003), an outcome one would also expect from loss of ARF function, since vesicle trafficking defects can cause accumulation of lipids and mis-sorted proteins. In addition to ARFs, we observed pronounced downregulation of parasite proteins involved in lipid metabolism upon NMT inhibition. For instance, multiple long-chain fatty acyl-CoA synthetases and other enzymes for fatty acid activation were diminished in amastigotes treated with DDD85646. These enzymes are not classical NMT substrates themselves, but their suppression likely reflects secondary effects: *T. cruzi* under NMT blockade may enter a state of lipid starvation or stress (due to membrane biogenesis defects), triggering a feedback reduction in lipid metabolic pathways. This is congruent with the idea that NMT inhibition has pleiotropic effects on parasite physiology (Herrera et al., 2016) by simultaneously wrecking vesicular transport, signaling, and metabolic processes, the parasite is pushed beyond compensatory limits. Although our current study did not investigate specific downstream pathways, previous reports suggest that TcNMT inhibitors may disrupt calcium signaling, ARF-mediated vesicular trafficking, and PKA-associated membrane localization, thereby impairing parasite differentiation and invasion. Indeed, our proteomic data also showed downregulation of several uncharacterized proteins containing N-terminal glycine motifs and enzymes involved in post-translational modifications (e.g., protein kinases and phosphatases) in DDD85646-treated parasites. While their specific roles remain to be elucidated, many of these probable represent additional myristoylated factors or downstream elements whose function depends on a correctly localized myristoylome. Intriguingly, one parasite protein, the calpain-like cysteine protease, was upregulated in trypomastigotes under NMT inhibition. Calpains are calcium-activated proteases linked to cytoskeletal remodeling and could be part of a stress response or differentiation signal, and their upregulation might indicate the parasites’ attempt to compensate for structural defects or to facilitate escape from the host cell under lethal duress (Roberts et al., 2014; Roberts and Fairlamb, 2016). This adaptive response, however, is insufficient to overcome the broad damage inflicted by loss of myristoylation. Although this study centers on biochemical, cellular, and proteomic analyses, subsequent research will integrate preclinical methodologies to comprehensively evaluate pharmacokinetics, therapeutic efficacy, and host safety in animal models. In summary, the biochemical and proteomic characterization presented here underscores the novelty and therapeutic potential of targeting N-myristoyltransferase (NMT) in *Trypanosoma cruzi*. Inhibiting TcNMT induces a cascade of disruptions within the parasite, halting vesicle trafficking, impairing lipid metabolism, and interfering with essential signaling pathways. These converging effects ultimately block parasite replication and differentiation. Such multifaceted disruption is particularly attractive in the context of drug development, as it reduces the likelihood of resistance emergence by imposing multiple lethal bottlenecks that would be difficult for the parasite to simultaneously evade. Importantly, NMT inhibition appears to act rapidly on the most pathogenic life stages of amastigotes and trypomastigotes, suggesting its potential to both clear acute infections and prevent progression to chronic disease. The stage-specific effects we observed further support the idea that NMT-directed therapies could be combined with, or sequenced alongside, existing drugs to maximize parasite clearance across the life cycle. Among the compounds tested, QUINE demonstrated moderate inhibitory activity against TcNMT and low cytotoxicity toward host cells, highlighting the need for further optimization to enhance its antiparasitic efficacy. Encouragingly, the moderate sequence divergence between parasites and human NMT (Herrera et al., 2016) offers a viable route for improving selectivity. Structure-guided design can exploit subtle differences in active site architecture to refine QUINE analogues for enhanced potency.

Furthermore, the distinct proteomic signatures elicited by TcNMT inhibition, including the depletion of important myristoylated proteins such as ARF1 and ARF3, may serve as pharmacodynamic biomarkers for confirming on-target activity in preclinical models. Together, these findings provide a strong rationale for advancing TcNMT as a drug target in Chagas disease. By bridging target-based discovery with mechanistic validation, this study lays the foundation for a new class of antitrypanosomal agents that exploit the parasite’s essential dependence on N-myristoylation.

While the current findings are promising, future work will address remaining limitations to strengthen the overall evidence base. Specifically, molecular dynamics simulations—offering greater resolution and reliability than docking alone, it will be employed to further validate and elucidate the predicted binding interactions between TcNMT and the identified *in silico* hits. Concurrently, the next phase will include comprehensive *in vitro* evaluations of these inhibitors, assessing their efficacy against additional *T. cruzi* strains, activity in diverse host cell types, cytotoxicity profiles, and potential synergy with existing antitrypanosomal agents. These studies will provide critical data to prioritize compounds for *in vivo* testing and guide the optimization of dosing regimens to maximize therapeutic windows.

## Data Availability

The mass spectrometry proteomics data have been deposited to the ProteomeXchange Consortium via the PRIDE partner repository with the dataset identifier PXD071927 and doi 10.6019/PXD071927.

## Supplementary material

SUPPLEMENTARY FIGURE S1 Dose-dependent effects of benznidazole and TcNMT inhibitors on AC16 human cardiomyocyte viability. AC16 cells were treated for 72 h with increasing concentrations of each compound, and viability was assessed using Hoechst 33342 and Propidium Iodide staining to distinguish live (blue bars) and dead (red bars) cells. Dose-response effects of in silico-selected TcNMT inhibitors: **(A)** DICY, **(B)** DANA, **(C)** QUINE, and **(D)** DDD85646. DDD exhibited the most potent activity, with significant parasite killing at sub-micromolar concentrations. Bars represent mean ± SD from three technical replicates. Statistical analysis was performed using one-way ANOVA with multiple comparisons (*p* < 0.05, p < 0.01, *p* < 0.001).

SUPPLEMENTARY FIGURE S2 Inhibition of Trypanosoma cruzi intracellular infection in AC16 human cardiomyocytes by TcNMT inhibitors. AC16 cells infected with *T. cruzi* (CL Brener strain) were treated for 72 h with increasing concentrations of TcNMT inhibitors and benznidazole (BZ; 100 μM, positive control). Infection levels were quantified by high-content imaging and expressed as the percentage of infected host cells. **(A)** DANA, **(B)** DICY, **(C)** QUINE, and **(D)** DDD85646 (DDD) were tested in serial dilutions (from 25 μM to 0.0007 µM). Benznidazole significantly reduced infection levels, and DDD showed the most consistent dose-dependent antiparasitic effect across the tested concentrations. Data represent mean ± SD from three technical replicates. Statistical significance was calculated relative to the infected untreated group using one-way ANOVA with Dunnett’s *post hoc* test (p < 0.05, p < 0.01, p < 0.001).

SUPPLEMENTARY FIGURE S3 Effect of TcNMT inhibitors on the intracellular replication of *Trypanosoma cruzi* amastigotes. AC16 human cardiomyocytes were infected with *T. cruzi* (CL Brener strain) and treated for 72 h with increasing concentrations of TcNMT inhibitors and benznidazole (BZ; 100 μM, positive control). After fixation and staining, the number of intracellular amastigotes per infected host cell was quantified using high-content imaging. **(A)** DANA, **(B)** DICY, **(C)** QUINE, and **(D)** DDD85646 (DDD) were evaluated in serial dilutions ranging from 25 μM to 0.0007 µM. Benznidazole significantly reduced intracellular replication. Among the test compounds, DDD showed the strongest and most consistent reduction in amastigote burden across multiple concentrations. Data represent the mean ± SD from three technical replicates. Statistical comparisons were made relative to the infected untreated group using one-way ANOVA with Dunnett’s *post hoc* test (p < 0.05, p < 0.01, p < 0.001).

SUPPLEMENTARY FIGURE S4 Effect of TcNMT inhibitors on the endocytic index of *Trypanosoma cruzi* in AC16 human cardiomyocytes. Infected AC16 cells were treated with increasing concentrations of TcNMT inhibitors or benznidazole (BZ; 100 µM) for 72 h. The endocytic index, calculated as the product of the percentage of infected cells and the average number of amastigotes per cell, was quantified by high-content imaging. **(A)** DANA, **(B)** DICY, **(C)** QUINE, and **(D)** DDD85646 (DDD) showed variable reductions in endocytic index across their respective concentration ranges (25 μM–0.0007 µM). Benznidazole and DDD demonstrated the most pronounced and consistent reductions. Data are presented as mean ± SD from three technical replicates. Statistical comparisons were made relative to the untreated infected control using one-way ANOVA with Dunnett’s *post hoc* test (p < 0.05, p < 0.01, p < 0.001).

SUPPLEMENTARY FIGURE S5 Proteomic profiling of DDD85646-treated samples reveals differential protein abundance in host and parasite compartments. Heatmap representation of significantly modulated proteins (adjusted p < 0.05) following treatment with the TcNMT inhibitor DDD85646 in **(A)** uninfected AC16 human cardiomyocytes, **(B)** intracellular amastigotes, and **(C)** extracellular trypomastigotes of *T. cruzi*. Protein abundance is shown as log_2_ fold change relative to untreated controls. The color scale indicates downregulation (purple) to upregulation (yellow). Notable changes include reduced expression of ADP-ribosylation factors, fatty acyl-CoA synthetases, OCRE domain-containing proteins, and phosphoinositide phospholipase C isoforms across parasite stages. Host cell modulation was comparatively limited but included MARCKS-related and signaling-associated proteins. Data represent the average from three independent biological replicates analyzed by LC-MS/MS.

SUPPLEMENTARY FIGURE S6 Sequence comparison of *T. cruzi* and *L. major* NMT. Residues that compose the binding site have been highlighted in green and are highly conserved.

SUPPLEMENTARY FIGURE S7 AlphaFold model of *T. cruzi* NMT. Both in the structure color code (left) and the heatmap (right), it can be observed that most regions have been modeled with very high (blue) or high (light blue) confidence level. The binding site rfor DDD85646 has been modeled with very high confidence.

SUPPLEMENTARY FIGURE S8 Superposition of the experimental structure of *L. major* NMT (cyan) and *T. cruzi* AlphaFold NMT model (magenta).

SUPPLEMENTARY FIGURE S9 Superposition of the experimental pose of DDD85646 and redocked pose (in *L. major* NMT), and the best docking pose in *T. cruzi* NMT.

SUPPLEMENTARY TABLE S1 Summary of differentially expressed proteins identified by LC-MS/MS analysis in DDD85646-treated and untreated samples, including uninfected AC16 cardiomyocytes, intracellular amastigotes, and extracellular trypomastigotes of T. cruzi. The table includes protein IDs, gene names, fold change (log2), p-values, adjusted p-values (FDR), and compartment-specific annotation.

SUPPLEMENTARY TABLE S2 Complete list of differentially expressed proteins identified by LC-MS/MS following DDD85646 treatment in uninfected AC16 cells, intracellular amastigotes, and extracellular trypomastigotes. The table includes protein identifiers, the organism of origin (human or T. cruzi), log2 fold change in abundance, p-values, false discovery rate (FDR)-adjusted p-values, and the specific experimental condition or compartment in which the differential expression was observed. This dataset represents the full proteomic response to TcNMT inhibition across host and parasite stages.

SUPPLEMENTARY TABLE S3 Differentially expressed proteins predicted or confirmed to be myristoylated in response to DDD85646 treatment across host and T. cruzi compartments. The table includes protein identifiers, organism of origin (human or parasite), predicted or validated N-myristoylation status, log2 fold change in abundance, p-value, and the cellular compartment where the protein was detected (AC16, intracellular amastigote, or trypomastigote). These findings highlight potential direct targets or downstream effectors of TcNMT inhibition.
