# Supplementary figures and images for: Advancing Trypanosoma cruzi N-myristoyltransferase as a drug target for Chagas disease through in silico discovery and biochemical evaluation
Source: Front Mol Biosci. 2026 Jan 6;12:1666768. doi: 10.3389/fmolb.2025.1666768 (PMC12816245; doi:10.3389/fmolb.2025.1666768)

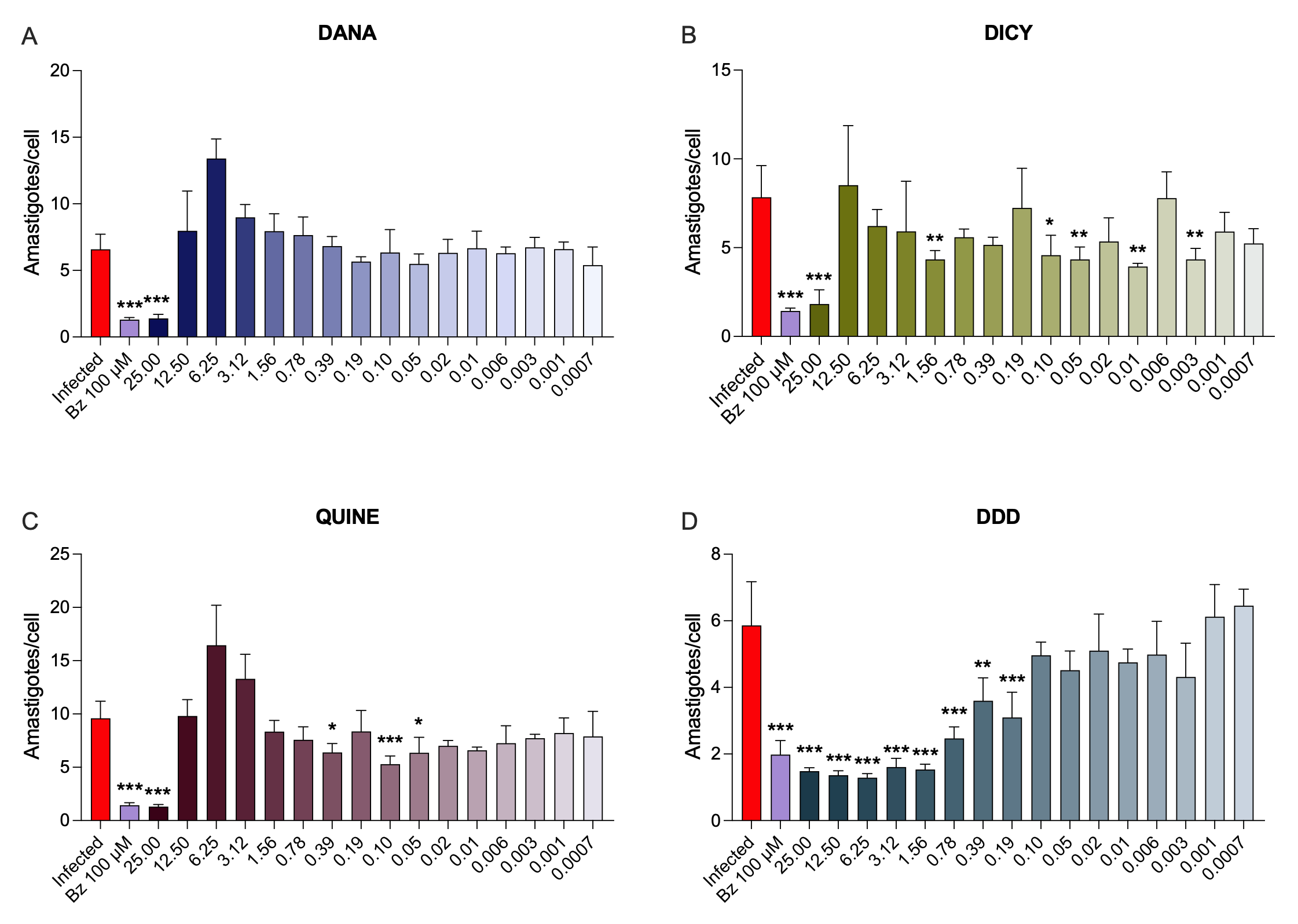

Supplement: Supplementary file 1 [file Image3.tiff]

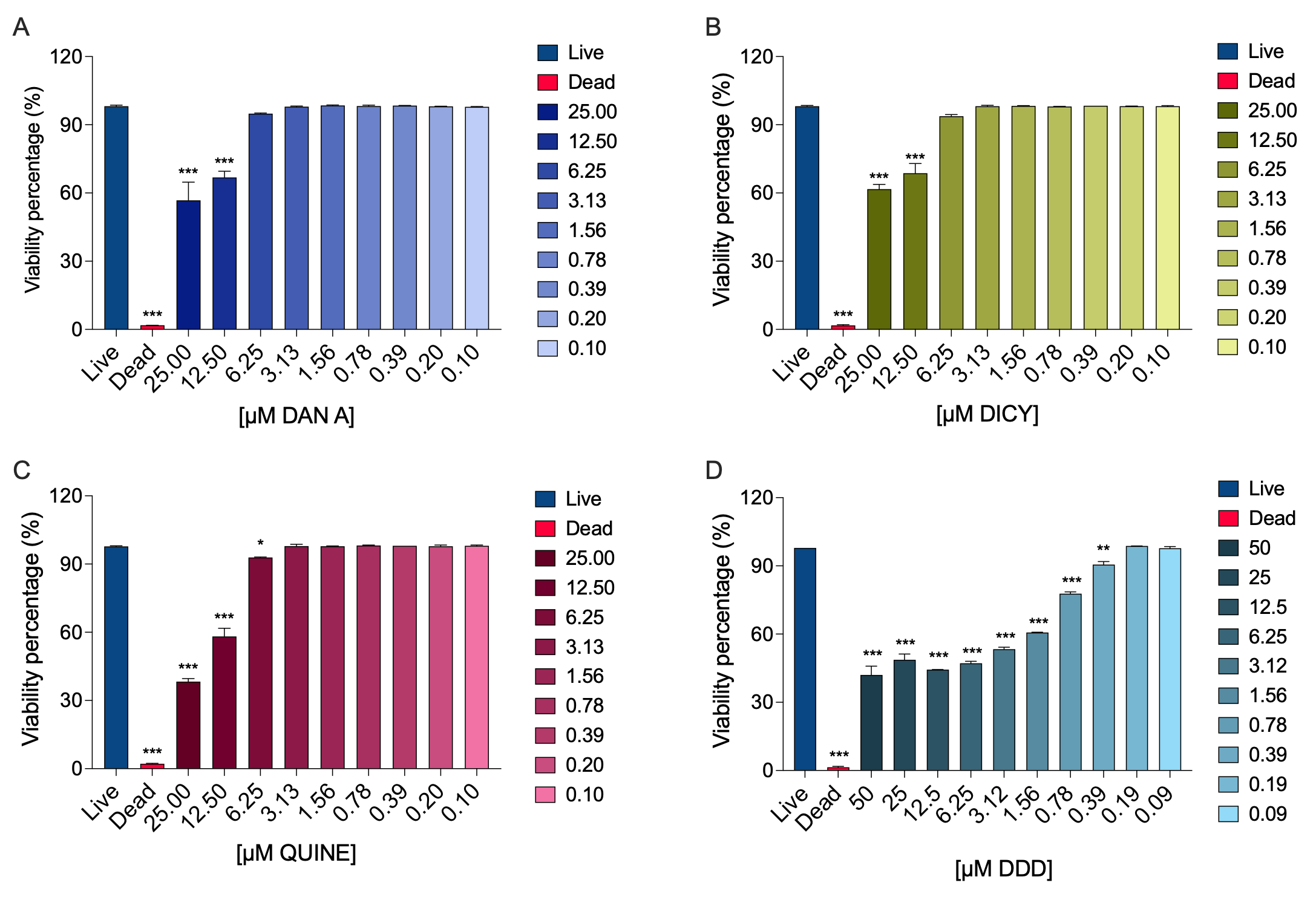

Supplement: Supplementary file 3 [file Image1.tiff]

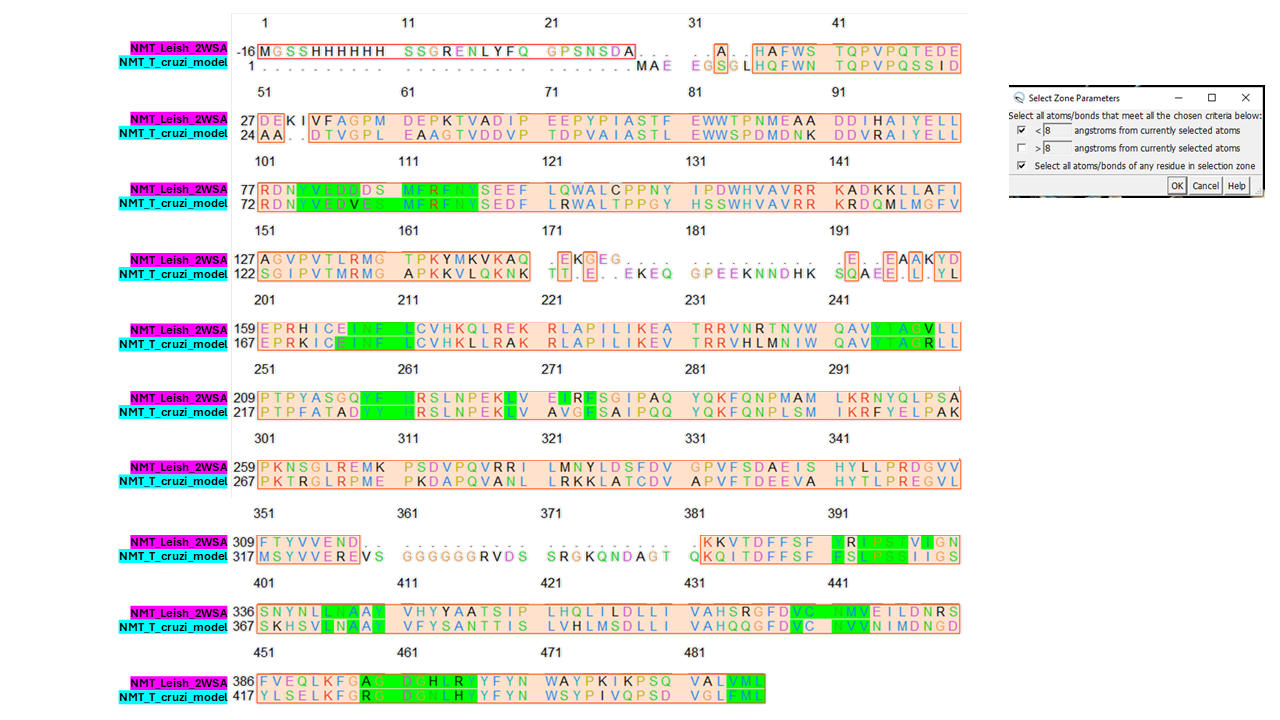

Supplement: Supplementary file 5 [file Image6.tif]

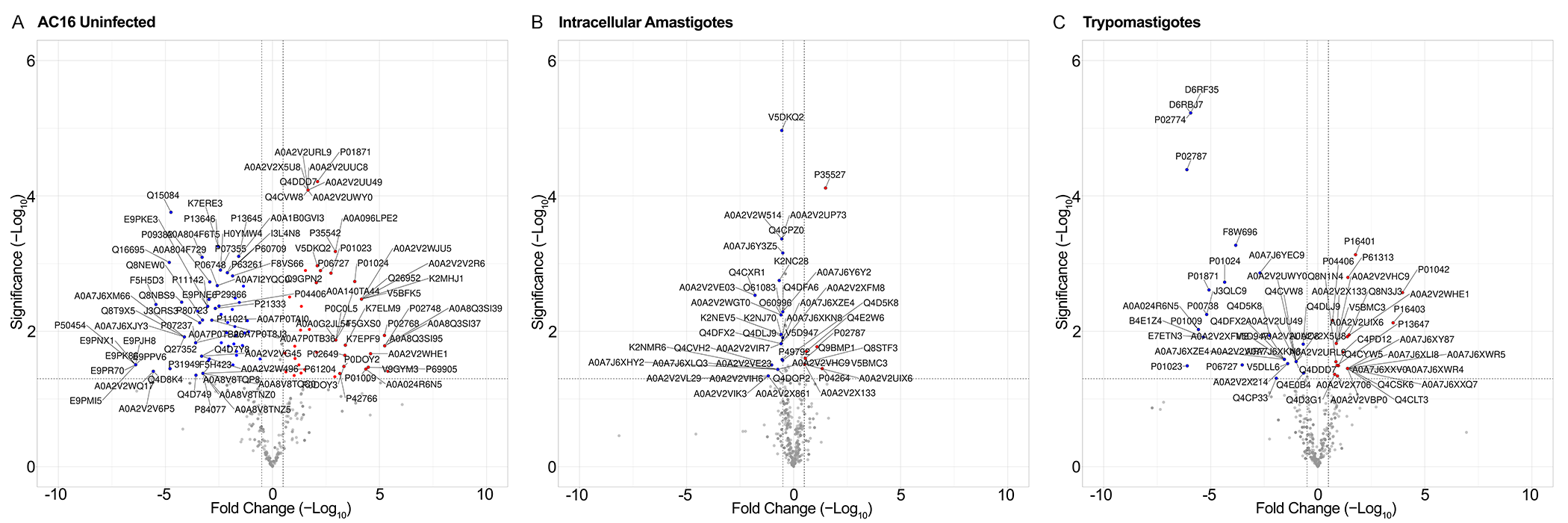

Supplement: Supplementary file 6 [file Image5.tiff]

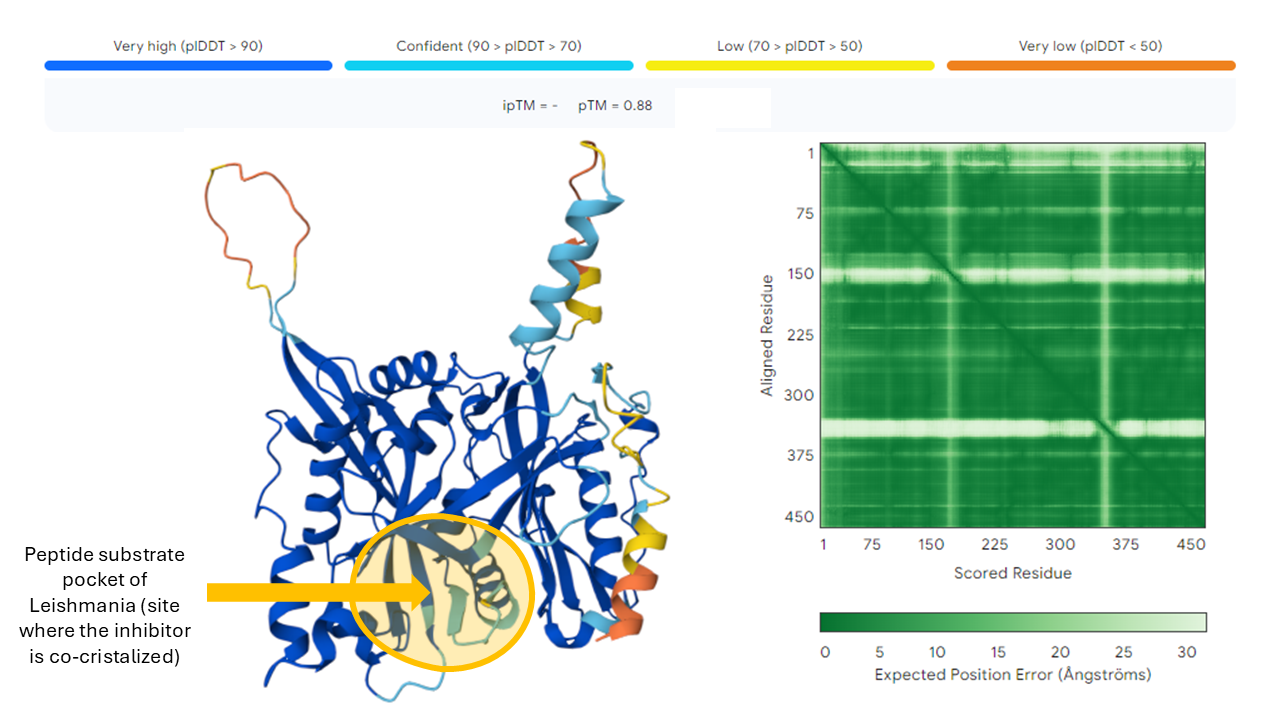

Supplement: Supplementary file 7 [file Image7.tif]

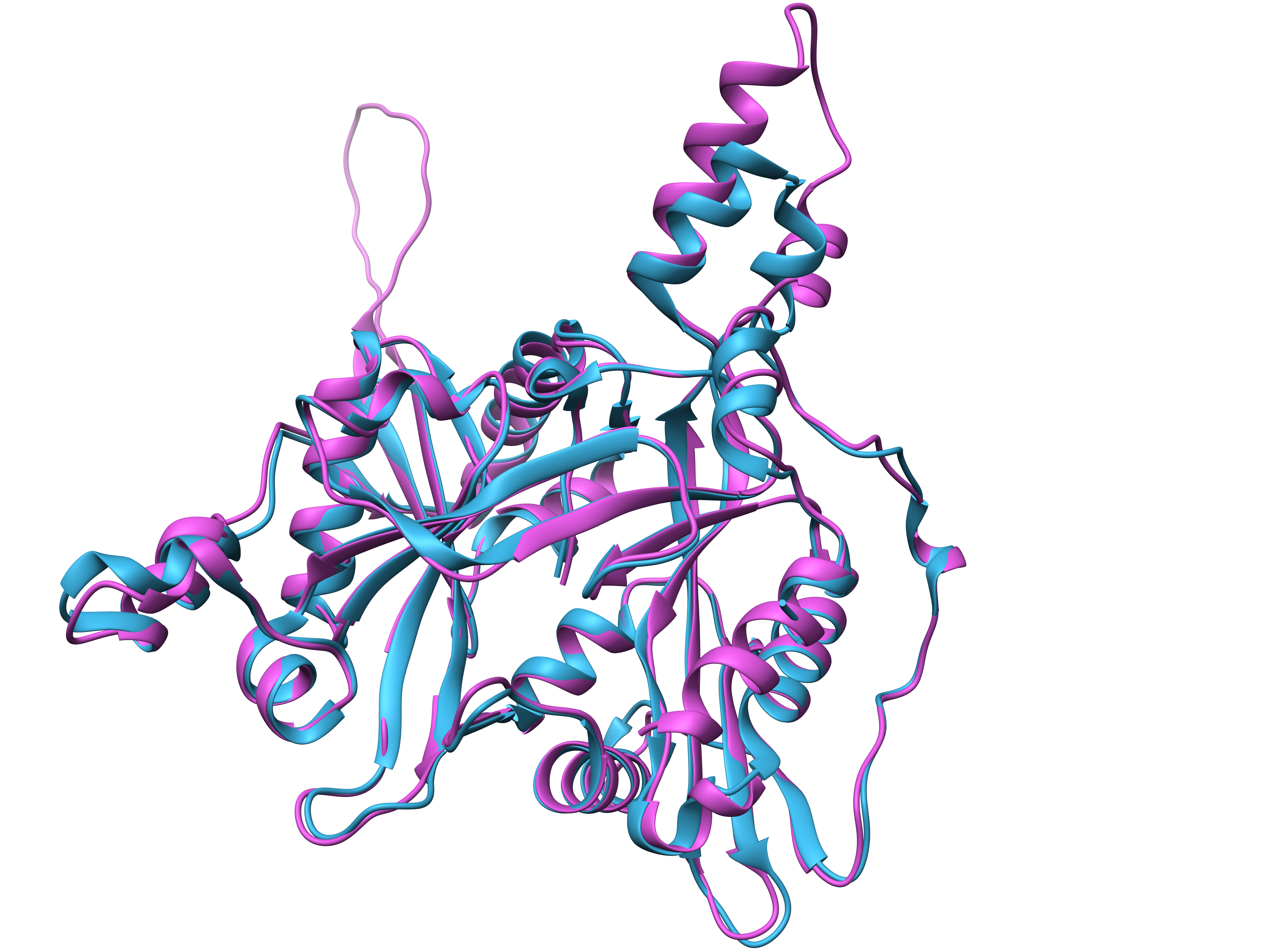

Supplement: Supplementary file 9 [file Image8.png]

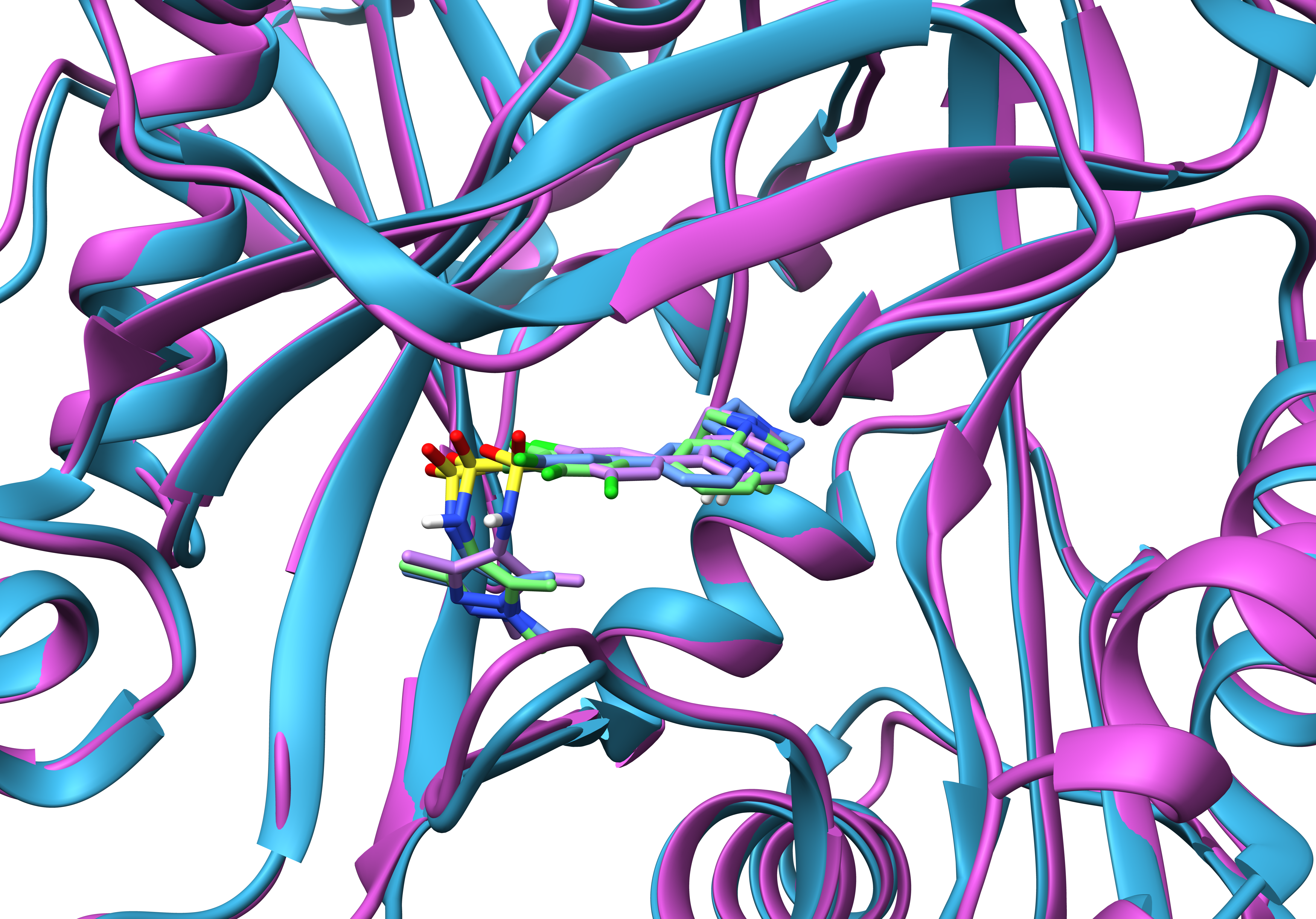

Supplement: Supplementary file 10 [file Image9.png]

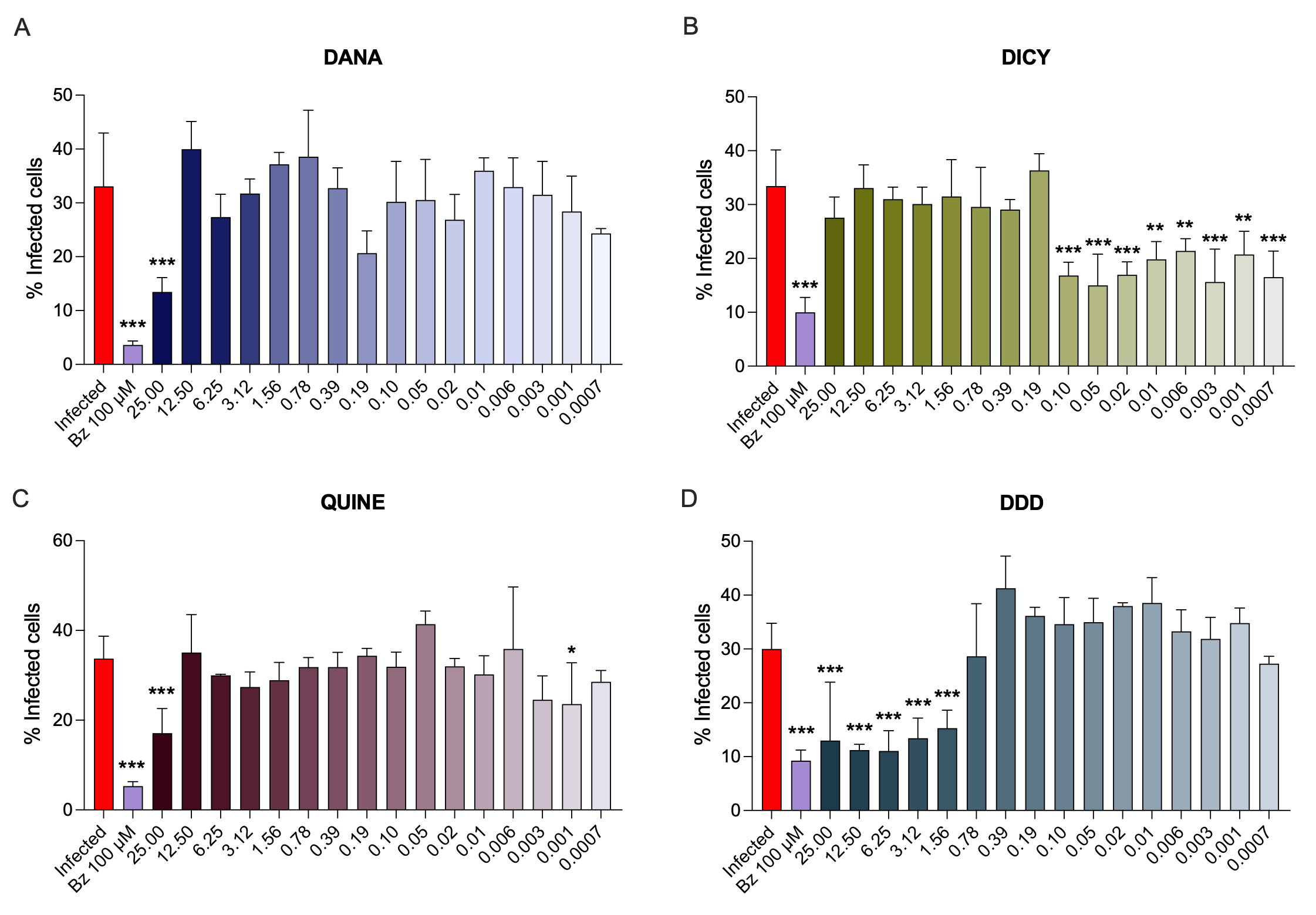

Supplement: Supplementary file 11 [file Image2.tiff]

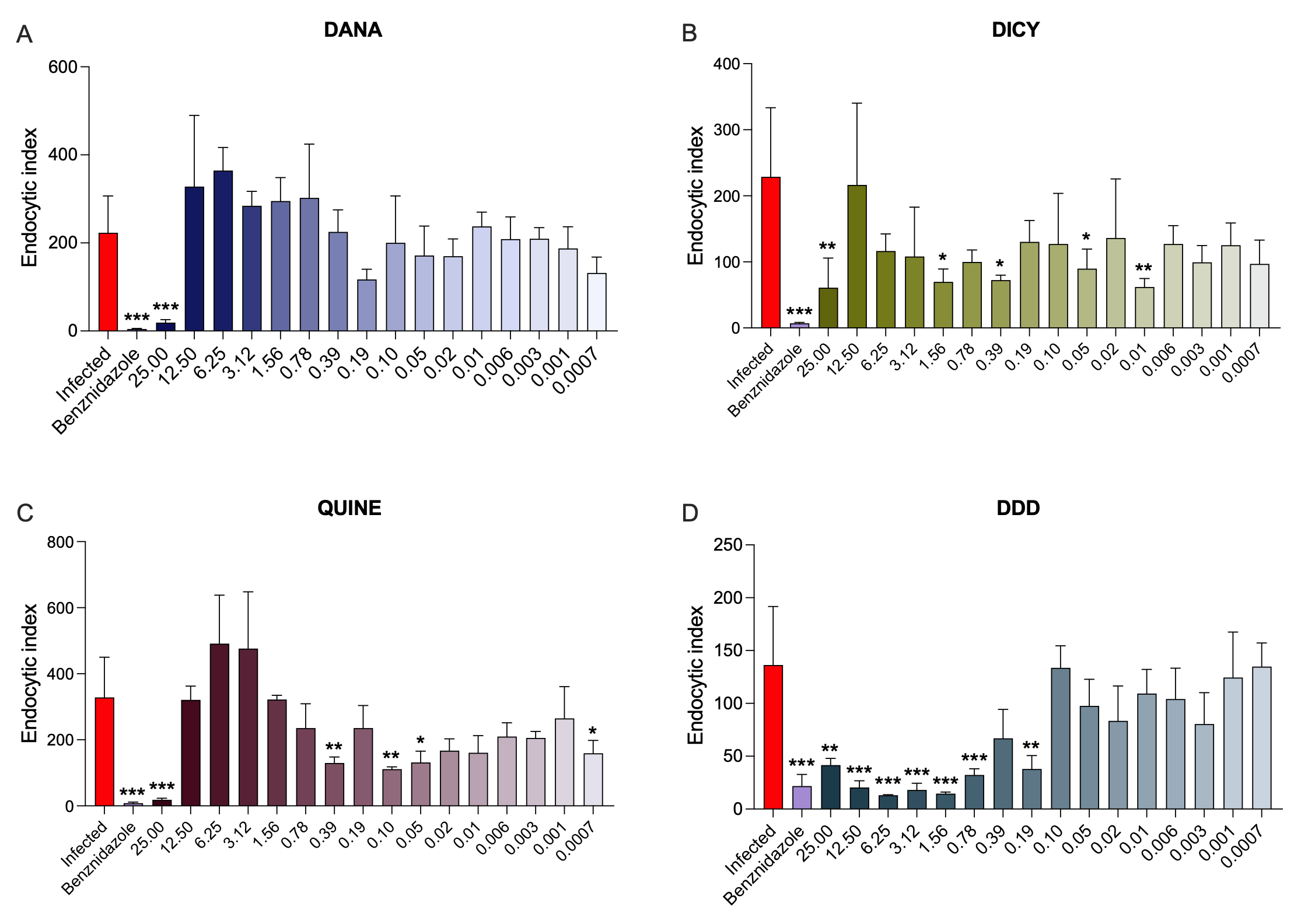

Supplement: Supplementary file 12 [file Image4.tiff]
